# Set of 15 SNP-SNP Markers for Detection of Unbalanced Degraded DNA Mixtures and Noninvasive Prenatal Paternity Testing

**DOI:** 10.3389/fgene.2021.800598

**Published:** 2022-02-10

**Authors:** Ranran Zhang, Yu Tan, Li Wang, Hui Jian, Jing Zhu, Yuanyuan Xiao, Mengyu Tan, Jiaming Xue, Fan Yang, Weibo Liang

**Affiliations:** ^1^ Department of Forensic Genetics, West China School of Basic Medical Sciences and Forensic Medicine, Sichuan University, Chengdu, China; ^2^ Department of Obstetrics and Gynecology, West China Second University Hospital, Key Laboratory of Birth Defects and Related Diseases of Women and Children of Ministry of Education, Sichuan University, Chengdu, China; ^3^ Department of Forensic Science and Technology, Sichuan Police College, Luzhou, China; ^4^ Department of Ultrasonography, West China Second University Hospital Sichuan University, Chengdu, China

**Keywords:** SNP-SNP microhaplotype, amplification-refractory mutation system PCR, SNaPshot, unbalanced and degraded mixture, cell-free fetal DNA, noninvasive prenatal paternity testing

## Abstract

Unbalanced and degraded mixtures (UDM) are very common in forensic DNA analysis. For example, DNA signals from criminal suspects are masked by a large amount of DNA from victims, or cell-free fetal DNA (cffDNA) in maternal plasma is masked by a high background of maternal DNA. Currently, detecting minor DNA in these mixtures is complex and challenging. We developed a new set of SNP-SNP microhaplotypes with short amplicons, and we successfully genotyped them using the new method of amplification-refractory mutation system PCR (ARMS-PCR) combined with SNaPshot technology based on a capillary electrophoresis (CE) platform. This panel reflects a high polymorphism in the Southwest Chinese Han population and thus has excellent potential for mixture studies. We evaluated the feasibility of this panel for UDM detection and noninvasive prenatal paternity testing (NIPPT). Fifteen SNP-SNPs detected minor DNA of homemade DNA mixtures, with a sensitivity of 0.025–0.05 ng and a specificity of 1:1,000. In addition, the panel successfully genotyped degraded DNA from single and mixed samples. Finally, 15 SNP-SNPs were applied to 26 trios. All samples displayed positive results with at least one marker to detect cffDNA. Besides, all fetal alleles in maternal plasma were confirmed by genotyping fetal genomic DNA from amniocentesis and paternal genomic DNA from peripheral blood. The results indicated that the SNP-SNP strategy based on the CE platform was useful for UDM detection and NIPPT.

## 1 Introduction

Unbalanced two-person DNA mixtures are frequently encountered in forensic cases (Tan et al., 2018). Effective isolation and genotyping of minor DNA is required and essential for forensic practice. However, degradation of DNA and unbalanced DNA mixtures make genotyping more difficult and challenging. The DNA fragments of degraded samples are usually 100–200 bp, including naturally degraded stains and cell-free fetal DNA (cffDNA) found in maternal plasma and other locations (Freire-Aradas et al., 2012). When an unbalanced mixed sample undergoes degradation, an unbalanced degraded mixture (UDM) is formed. Many problems occurred while detecting the UDM, such as poor sample recovery, stutter artifacts, unbalanced peak heights, allele dropouts, and complicated interpretation of the results.

A typical UDM is the cell-free DNA (cfDNA) in maternal blood circulation, which is a mixture of the main maternal DNA derived from the maternal hematopoietic system (Scotchman et al., 2019) and fetal DNA released by the apoptosis of embryonic cytotrophoblasts (Alberry et al., 2007). Due to its apoptotic nature, cffDNA consists mainly of short fragments with a median length of 143 bp (Alberry et al., 2007; Lo et al., 2010). cffDNA accounts for approximately 5–20% of the total cfDNA, with an upward trend evident throughout pregnancy (Scotchman et al., 2019). These features make the noninvasive prenatal paternity testing possible. However, the low level of cffDNA in maternal plasma challenges traditional detection methods. For example, STR and SNP markers based on PCR and CE are not sensitive enough to detect the components accounts for less than 20% of mixtures (Wang et al., 2012; Tan et al., 2017b). The fragment size of cffDNA is mostly 100–200 bp and rarely longer than 250 bp, which excludes most STR loci. In addition, stutter products interfere the analysis of cffDNA. SNP, as a single base variation and no stutter products present during the PCR amplification, is considered more appropriate for NIPPT (Chang et al., 2019; Tam et al., 2020). However, the biallelic nature of SNP results in limited polymorphism at a single locus. Thus, more loci are needed to construct a detection panel with a reasonable discrimination ability.

In view of the problems encountered during UDM genotyping, forensic scientists have developed many new methods to detect these kinds of mixtures. For example, deletion insertion polymorphism (DIP)-STR and single nucleotide polymorphism (SNP)-STR are two new types of compound genetic markers. The DIP or SNP is accompanied by a closely linked STR. DIP-STR and SNP-STR markers could reach a mixture ratio of 1:1,000 and showed potential in detecting cffDNA in maternal plasma. However, the absence of multiplex reactions required more DNA templates. Furthermore, more loci were needed to improve the panel’s identification power (Tan et al., 2017b; Wang et al., 2017; Cereda et al., 2018; Tan et al., 2018). DIP-SNP is also a novel compound genetic marker that consists of a DIP and a tightly linked SNP. An analysis of 34 DIP-SNPs revealed that these loci improved the sensitivity of mixtures and degraded samples. However, the lower DIP frequency and the longer amplicon of DIP-SNP resulted in a low probability of revealing an effective genotype of the degraded mixtures (Liu et al., 2018; Liu et al., 2019). Therefore, the recovery of DNA genotypes from UDM remains a challenge.

Microhaplotypes are a novel compound genetic marker that contains two or more closely linked SNPs (within 200 bp). The marker was firstly developed by Kidd et al. (2013). Compared with STRs, microhaplotypes do not contain repeated motifs and thus do not cause stutter products. Furthermore, compared to SNPs, microhaplotypes are more polymorphic. Therefore, microhaplotypes combine the advantages of STRs and SNPs, which suggests good potential in genotyping UDM.

We previously developed a set of 15 new SNP-SNPs and used ARMS-PCR combined with SNaPshot technology to construct a panel based on the CE platform (Zhang et al., 2020). In addition, the genotyping of 155 Southwest Chinese Han individuals revealed a high discrimination ability and a good polymorphism of the panel and its potential for mixture detection. The amplicon of each locus was about 60–150 bp, which was shorter than DIP-STRs (Castella et al., 2013; Oldoni et al., 2015; Tan et al., 2017b; Oldoni et al., 2017), SNP-STRs (Wang et al., 2015; Tan et al., 2017a; Wang et al., 2017; Tan et al., 2018; Wei et al., 2018), and DIP-SNPs (Liu et al., 2018; Liu et al., 2019) reported before. In this study, we applied these SNP-SNPs to homemade degraded single and mixed samples and 26 cffDNA samples. The results demonstrated that this method can effectively detect UDM and can be used for NIPPT.

## 2 Materials and Methods

### 2.1 Sample Collection and Extraction

#### 2.1.1 Peripheral Blood Samples

A total of 32 peripheral blood samples of Southwest Chinese Han individuals with DNA concentrations over 50 ng/μL extracted by phenol-chloroform were quantified using a NanoDrop™ 1,000 spectrophotometer (Thermo Fisher Scientific, Waltham, MA, United States). We ensured that our work was carried out in accordance with the ethical code of the World Medical Association (Declaration of Helsinki).

#### 2.1.2 Degraded DNA Samples

To obtain the artificial single-source degraded DNA, the DNA with the highest heterozygosity from the 32 samples was incubated at 98°C for 35, 40, and 45 min in the Eppendorf 6331 Nexus Gradient Flexlid Thermal Cycler (Eppendorf, Hamburg, Germany). In addition, the standard DNA M308 was incubated in a PCR system at 98°C for 120, 160, and 170 min.

To obtain artificially mixed and degraded DNA, the two DNA samples with the highest degree of heterozygosity and homozygosity from the 32 samples were mixed and then subjected to a series of dilutions (1:1, 1:10, 1:20, 1:50, 1:100, 1:500, and 1:1,000) and incubated at 98°C for 35, 40, and 45 min.

The degree of DNA degradation after incubation was determined using a High Sensitivity DNA Kit on Agilent 2100 Bioanalyzer (Agilent Technologies, Santa Clara, CA, United States) and the AGCU EX22 Kit (Applied ScienTech, Jiangsu, China) on an ABI 3500 Genetic Analyzer (Applied Biosystems), according to the manufacturers’ instructions. The results were analyzed using the GeneMapper ID-X v1.2 software (Applied Biosystems).

#### 2.1.3 Parental Peripheral Blood and Cell-Free Fetal DNA Samples

As approved by the Ethics Committee of Sichuan University (KS2019042), the criteria for selecting volunteers were: (1) singleton pregnancy; (2) pregnant women whose weight should be less than 100 kg; and (3) pregnant women with or without a history of cancer (Tan et al., 2021). Ten milliliters of peripheral venous blood were collected from 26 pregnant women over 18 weeks. EDTA was added to prevent coagulation. For verification, venous blood (1.5 ml) of the fetus’s biological father and the mother’s amniotic fluid sample (2 ml) were collected. Written informed consent was obtained from all the participants.

The maternal plasma was separated by the two-step centrifugation (Shea et al., 2013). In the first step, 10 ml of peripheral venous blood from the pregnant women was centrifuged at 1,600 g for 10 min at 4°C. After the first centrifugation, plasma and leukocytes were collected separately in collection tubes. The plasma recovered in the first step was centrifuged for the second time at 16,000 g at 4°C for 10 min. According to the manufacturer’s instructions, the MagMAX cfDNA Isolation Kit (Thermo Fisher Scientific) was used to extract free DNA from the collected plasma. The whole blood genomic DNA (gDNA) of each mother and father was isolated using a commercial kit (BioTeke, Beijing, China). The fetal DNA was extracted from amniotic fluid samples using a DNeasy Blood & Tissue kit (QIAGEN, Hilden, Germany). The concentrations of cfDNA and fetal DNA extracted from the amniotic fluid sample were quantified using a Qubit™ dsDNA HS Detection Kit (Thermo Fisher Scientific). The genomic DNA of the parents was quantified using a NanoDrop™ 1,000 spectrophotometer (Thermo Fisher Scientific). The extracted DNA samples were stored at −20°C until used.

### 2.2 Analysis of SNP-SNPs Sensitivity

For each SNP-SNP, samples whose SNP1 genotype were heterozygous were selected. Then these single source samples were diluted at concentrations of 1, 0.5, 0.1, 0.05, and 0.025 ng/μL. Two allele-specific primers of the locus, i.e., F1 and F2 primers, were used to amplify samples at different concentrations. The amplified products were then used as the DNA template of the single base extension (SBE) for the SNaPshot reaction.

For a single locus of common samples, ARMS-PCR was followed by 30 cycles of 94°C for 30 s, 57.8°C for 90 s, and 72°C for 60 s, and a final extension at 60°C for 30 min. SBE involved 25 cycles of 96°C for 10 s, 53°C for 5 s, and 60°C for 30 s in the Eppendorf 6331 Nexus Gradient Flexlid Thermal Cycler (Eppendorf Scientific). A total of 1 μL of template DNA was used. The details of the reaction system and the parameters were reported before (Zhang et al., 2020).

For multiple loci of the common samples, except for the increase in SBE cycles, the rest of the reaction system and parameters were the same as detailed above. The SBE was performed using 28 cycles.

For cffDNA detection using single locus, the reaction system and parameters were the same as above, except for the increase in ARMS-PCR and SBE cycles. The ARMS-PCR and SBE were both performed using 32 cycles.

### 2.3 Analysis of SNP-SNPs Simulated Mixture

For the single reaction, samples with homozygous SNP1 were used as the major component and samples with heterozygous SNP1 were used as the minor component to construct a two-person DNA mixture. The DNA amount of the minor component in the mixture was fixed at 0.05 ng. Then different amounts of the major component DNA were added to the minor component to form the mixtures with ratios of 1:1, 1:10, 1:20, 1:50, 1:100, 1:500, and 1:1,000. A total of 1 μL of each mixture was collected and analyzed to evaluate the efficiency of the single system.

For the multiplex reaction, two samples with relative more effective loci were selected. And mixtures were mainly formed by two patterns: (1) the major and minor components were oppositely homozygous for SNP1; (2) the major was homozygous and the minor was heterozygous for SNP1, respectively. Finally, the mixture ratios and the analysis were performed as described to evaluate the efficiency of the multiplex system.

### 2.4 Analysis of SNP-SNPs Simulated Degradation of DNA

We used the commercial AGCU EX22 STR kit (Applied ScienTech) and the 15 SNP-SNP multiplex system (Supplementary Table S1) to genotype the simulated degraded single-source and degraded mixed DNA samples and evaluate the ability of both in detecting degraded DNA.

### 2.5 SNP-SNPs Detection of Cell-Free Fetal DNA

**FIGURE 1:**
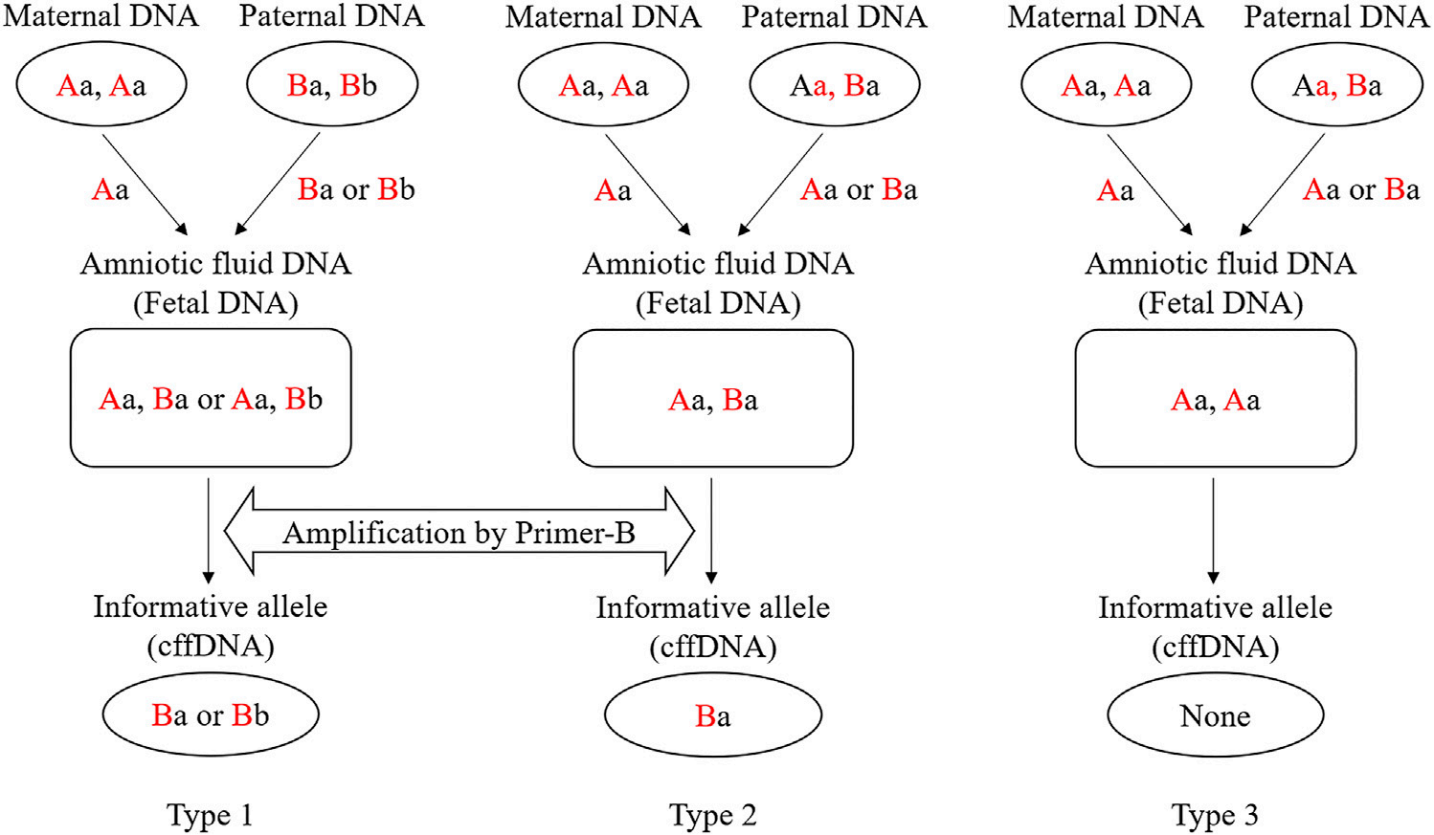
Three microhaplotype patterns (Types 1, 2, and 3) in a cfDNA mixture and examples of genotype combinations of parents and fetus. Red capital letters indicate the SNP1 genotypes (“A” can be any of A/G/C/T), and black lowercase letters indicate the SNP2 genotypes (“a” can be any of A/G/C/T). For the SNP1 in Type 1, the mother and father carry different SNP alleles. Therefore, no matter which allele (Ba or Bb) of the father is passed to the fetus, the corresponding allele-specific primer can be used for targeting (Primer-B). For the SNP1 in Type 2, the father is heterozygous. Then the fetal DNA can only be recognized when the allele (Ba) inherited from the father is not shared with the mother. For the SNP1 in Type 3, the father is heterozygous. When the allele (Aa) inherited from the father is shared with the mother, the fetal DNA cannot be recognized.

Reference DNA samples and amniotic fluid samples of 26 families were genotyped using the 15 SNP-SNP multiplex system to determine the informative markers for plasma DNA analysis. Specifically, there were two genotype combinations which allele-specific primers can be applied to. For Type 1 (Figure 1), the mother and father were opposite SNP1 homozygotes. For Type 2 (Figure 1), the mother was homozygous for SNP1, and the father heterozygous for SNP1. As a result, the alleles of SNP1 inherited from the father in the fetus were different from those inherited from the mother, that is, alleles were not shared. The targeted fetal allele which is different from the mother is called informative allele, and the corresponding markers are called informative markers. However, for Type 3 (Figure 1), no informative markers can be used to target the fetal DNA. For these samples with informative alleles, Type 1 and Type 2, the informative markers were used to detect cffDNA in maternal plasma, and a single reaction was performed for each informative marker. The products were separated by CE on a 3130 Genetic Analyzer (Applied Biosystems). To isolate the DNA fragments, 1 μL of SBE products was mixed with 9 uL of GeneScan-120 LIZ™ internal size standard (Applied Biosystems) and Hi-Di formamide (Applied Biosystems; Liz120:Hi-Di = 1:100). The results were analyzed using the GeneMapper ID v3.2 software (Applied Biosystems). The detection thresholds of the peak height in different colors (green, blue, black, and red) and internal standards were set to 50 relative fluorescence units by default.

## 3 Results

### 3.1 Sensitivity Efficiency

**FIGURE 2:**
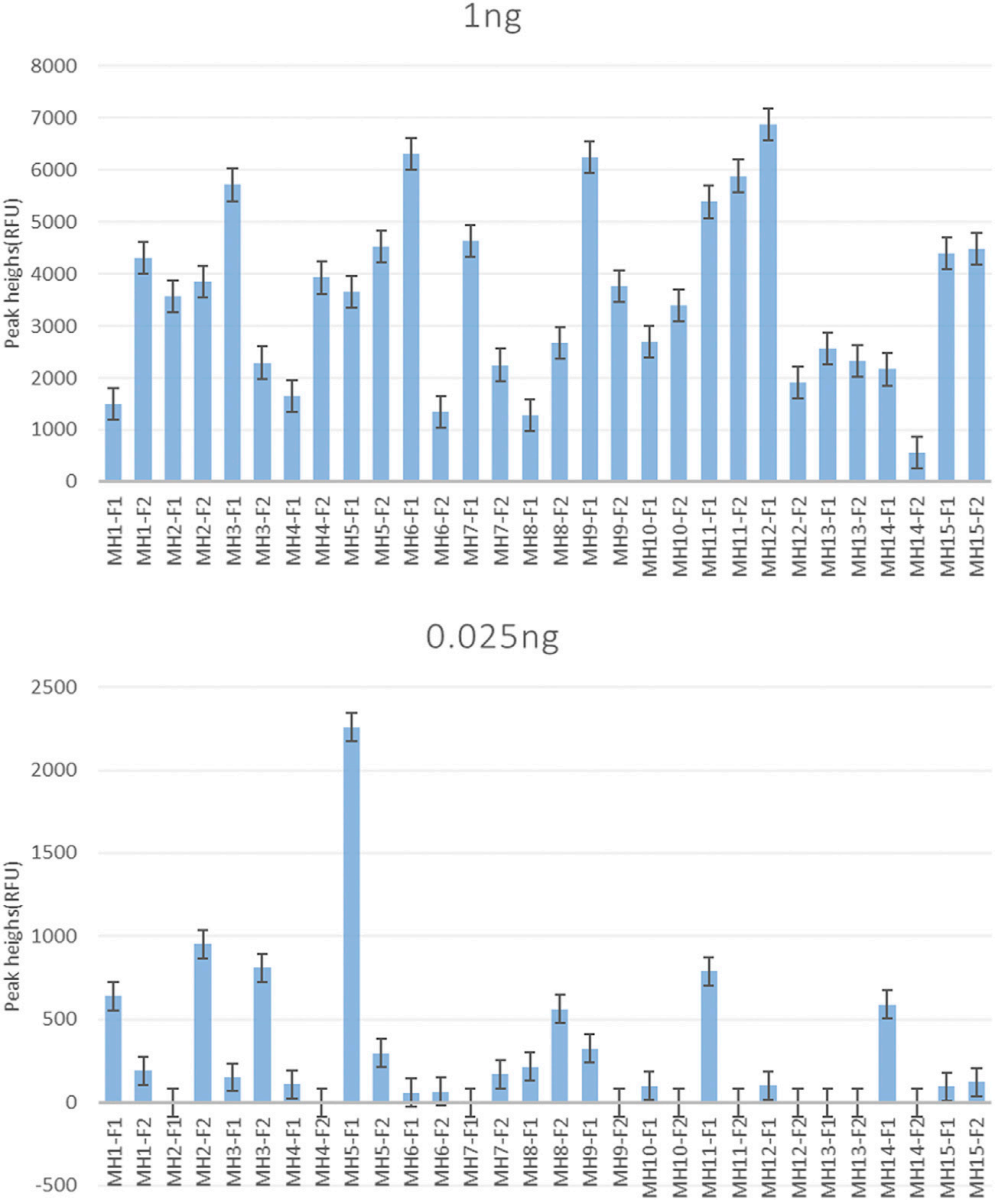
Sensitivity of the single allele-specific primer. MH1∼MH15 correspond to 15 SNP-SNPs markers. Also, F1 and F2 represent the two forward allele-specific primers for each locus. The results show the peak height of each primer using 1 and 0.025 ng of the template DNA. Except for MH2-F1, MH4-F2, MH7-F1, MH9-F2, MH10-F2, MH11-F2, MH12-F2, MH13-F1/F2, and MH14-F2, which had a detection sensitivity of 0.05 ng, and the other primers showed a positive result of 0.025 ng.

For the single reaction, all 30 allele-specific primers, except the detection sensitivities of MH2-F1, MH4-F2, MH7-F1, MH9-F2, MH10-F2, MH11-F2, MH12-F2, MH13-F1/F2, and MH14-F2 was 0.05 ng (MH stands for microhaplotype, and the number after MH was used to distinguish different loci. F1 and F2 represent the two allele-specific primers, respectively). The other 20 primers obtained positive results when the template DNA was as low as 0.025 ng (Figure 2). Supplementary Figure S1 displays the specific profiles.

For multiplex reactions, the sensitivity of random DNA samples and standard DNA 9947A reached 0.0625 ng. The sensitivity of 9947A was 0.03125 ng (Zhang et al., 2020).

### 3.2 Detection Efficiency of Simulated Mixtures

SNP-SNP genotypes are based on specific primers targeting each SNP1 allele, regardless of the SNP2 genotype. Therefore, when using the minor allele-specific primers to amplify the two-person mixture, only the minor allele was amplified in the simulated mixture.

**FIGURE 3:**
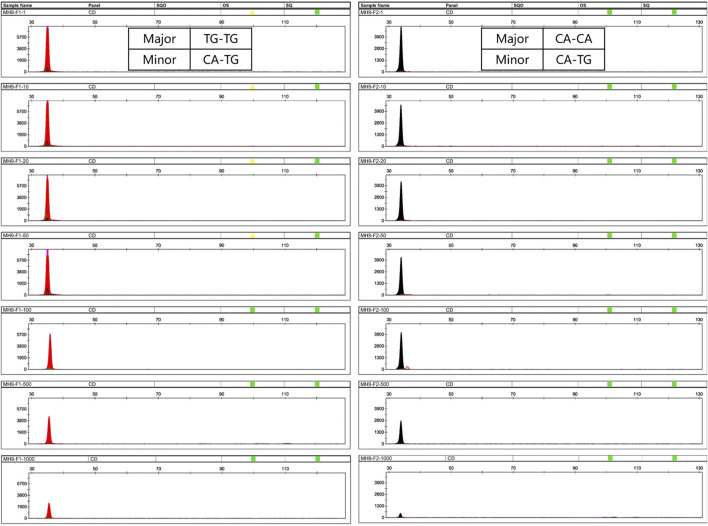
Profiles of mixtures using a single SNP-SNP marker. Here, MH9 was selected as an example. **(A,B)** represent two different mixtures. In mixture A, the minor DNA genotype is CA-TG, the genotype of the major DNA is TG-TG, and the allele-specific primer MH9-F1 was used to target CA of the minor DNA. Similarly, in mixture B, the minor DNA genotype is CA-TG, the genotype of the major DNA is CA-CA, and the primer MH9-F2 was used to target the TG of the minor DNA. The minor DNA was fixed at 0.05 ng, the mixing ratio was 1:1–1:1,000, and the template DNA amount was 1 μL. At this locus, the two allele-specific primers can successfully amplify minor DNA at a ratio of up to 1:1,000.

For the single reaction, all allele-specific primers successfully targeted the minor DNA with a ratio up to 1:1,000, except for MH1-F2, MH7-F1, and MH13-F2, which there were no samples detected homozygous for SNP1. For mixtures, the DNA input was 1 μL (the minor component was fixed at 0.05 ng). In addition, as the ratio changed from 1:1 to 1:1,000, the peak heights of the minor alleles gradually decreased (Figure 3).

**FIGURE 4:**
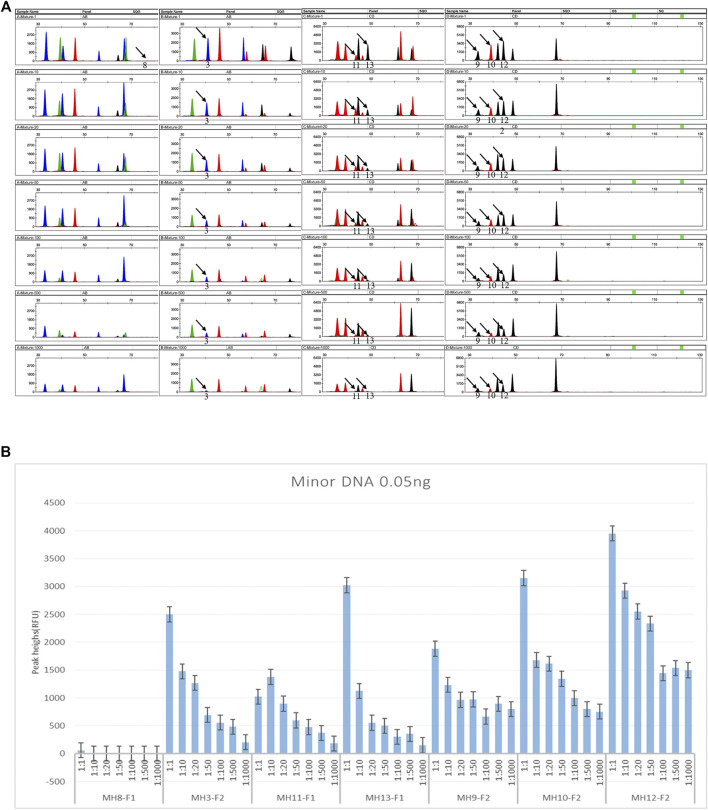
Detection sensitivity of DNA mixtures (input amount 1 μL). **(A)** Electropherograms of the mixture sensitivity. The simulated mixture was made from two randomly selected individuals. Seven loci were informative markers. For MH8, the minor DNA genotype is AA-CG, and the major is CG-CG. For MH3, the minor DNA genotype is TC-TC, and the major is CC- CC. For MH11, the minor DNA genotype is CA-TG, and the major is TG-TG. For MH13, the minor DNA genotype is CG-TG, and the major is TG-TG. For MH9, the minor DNA genotype is CA-TG, and the major is CA-CA. For MH10, the minor DNA genotype is CT-GT, and the major is CT-CT. For MH12, the minor DNA genotype is CG-TG, and the major is CG-CA. The black arrow indicates the informative allele of minor DNA. The detection ratio of minor DNA alleles was as high as 1:1,000. As the ratio increased, the specific peaks from minor contributors tended to decrease or even disappear. Note that as the ratio increased, the detection sensitivity of mixtures at the seven loci was different. Except for the minor specific alleles (AA) at MH8, which were only detected in 1:1 (0.05 ng minor DNA and 0.05 ng major DNA), the minor specific alleles at the remaining six loci could be detected in the 1:1,000 ratio. **(B)** Summary of the peak heights of the minor DNA informative markers in a series of dilutions. The homemade two-person mixture was diluted sequentially to 1:1, 1:10, 1:20, 1:50, 1:100, 1:500, and 1:1,000. The minor components were fixed at 0.05 ng. Using the multiplex PCR-CE method, up to 1:1,000 minor DNA could be detected among the seven SNP-SNPs.

For the multiplex system, to explore the detection threshold of the minor DNA in the mixture, a homemade two-person mixture was genotyped after a series of dilutions from 1:1 to 1:1,000. The minor DNA was fixed at 0.05 ng. Three common informative loci were present. SNP1 is an opposite homozygote (AA, BB), one was homozygous, and one was heterozygous (AA/BB, AB). Seven SNP-SNPs (MH8, MH3, MH11, MH13, MH9, MH10, MH12) were detected as informative markers (Figure 4). The minor genotypes were AA-CG, TC-TC, CA-TG, CG-TG, CA-TG, CT-GT, and CG-TG. The major genotypes were CG-CG, CC-CC, TG-TG, TG-TG, CA-CA, CT-CT, and CG-CA. For the multiplex reaction, except for MH8 with minor DNA detected only in the 1:1 mixture, the remaining six loci could detect minor DNA in ratios of 1:1 to 1:1,000.

### 3.3 Detection Efficiency of Simulated Degraded DNA

The degradation degree of the samples is shown in Supplementary Figure S2. Most of the fragments were concentrated below 200 bp. Supplementary Figures S3A,B showed that when random individual DNA was treated at 98°C for 35, 40, and 45 min, long-segment STR genotyping failed. When the standard DNA M308 was treated at 98°C for 120, 160, and 170 min, the long-segment STR genotyping also failed. In contrast, all SNP-SNPs in the artificially degraded samples were successfully genotyped (Supplementary Figures S3C,D). These results showed that SNP-SNPs were more effective in genotyping degraded DNA, which may be because of the higher specificity of primers and smaller amplicons. Supplementary Figure S4A shows the STR genotypes of two-person DNA incubated at 98°C for 35, 40, and 45 min. Supplementary Figure S4B shows the corresponding SNP-SNP genotypes. The STRs failed to genotype, but all SNP-SNPs were successfully genotyped.

### 3.4 Informative Markers Based on DNA Genotypes of Reference Families

An informative marker refers to a marker which the fetal SNP1 allele is inherited from the father and is not shared with the mother. The unique fetal allele inherited from the father in maternal plasma is the target sequence of allele-specific primers. According to the different genotype combinations of the parents, the informative markers are divided into two types (Type 1 and 2). As shown in Figure 1, the father’s SNP1 in Type1 is homozygous and not shared with the mother. Regardless of which allele is inherited, the haplotype passed on from the father to the fetus can be found in the mother’s plasma. The father’s SNP1 in Type 2 was heterozygous. When the haplotype inherited from the father carries the SNP1 allele that is not shared with the mother, fetal DNA can be identified from the mother’s plasma. However, in Type 3, the father’s SNP1 was heterozygous and the haplotype inherited from the father was the same with the mother at SNP1. Therefore, no fetal DNA could be identified from the mother’s plasma.

**FIGURE 5:**
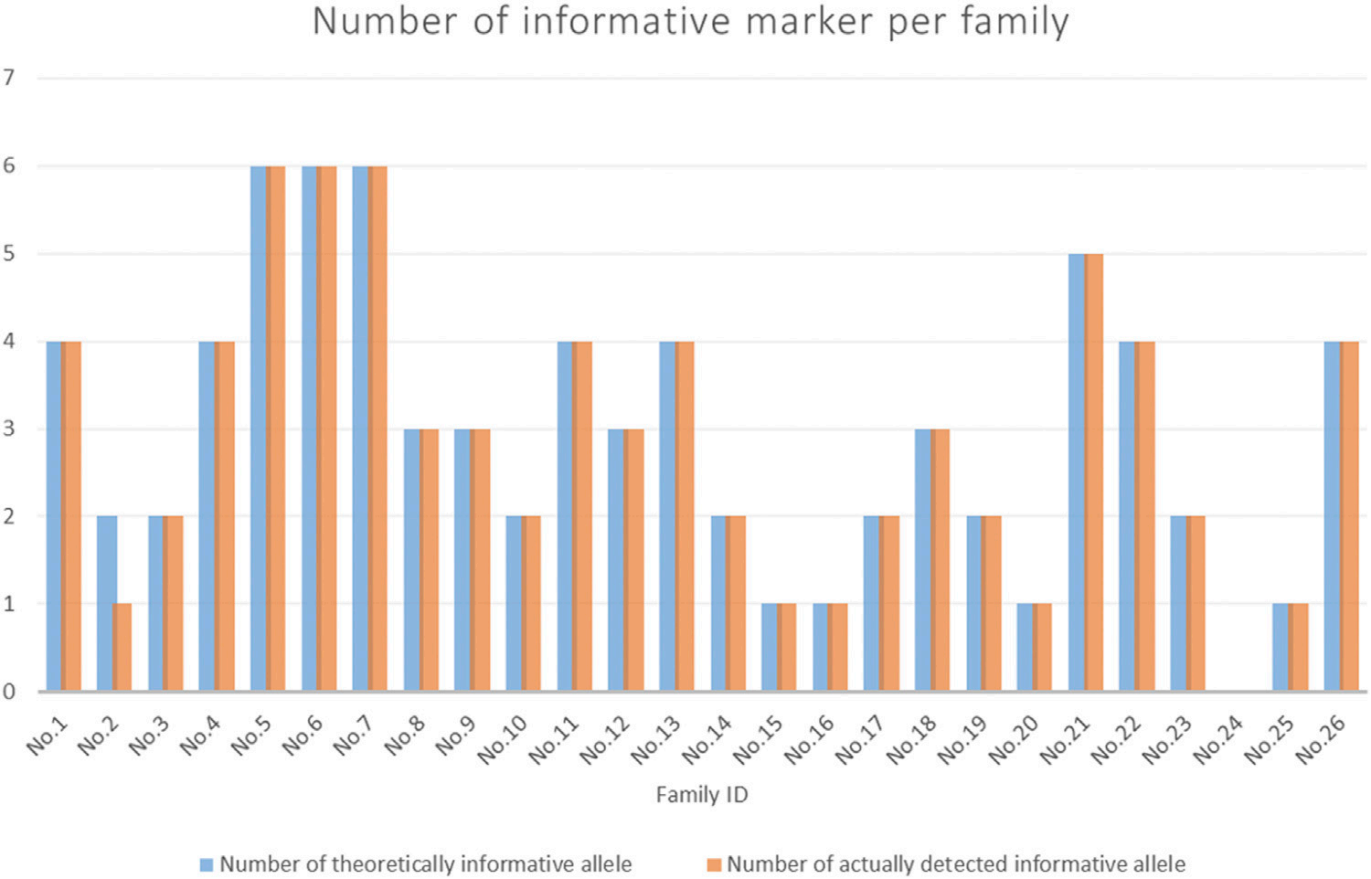
The number of informative markers for each family. The blue bars showed the theoretical number of informative markers (expected). The orange bars indicated the actual number of informative markers detected (observed).

The participants’ data are shown in Supplementary Table S2. Details of the informative markers for each family are shown in Supplementary Table S3. Figure 5 shows the number of informative markers for each family. The total number of informative markers in each family ranged from 0 to 6. Three families (No. 5∼7) displayed the most informative marks (*n* = 6), while family No. 24 displayed no informative markers.

### 3.5 Detection of Cell-Free Fetal DNA Informative Markers

**FIGURE 6:**
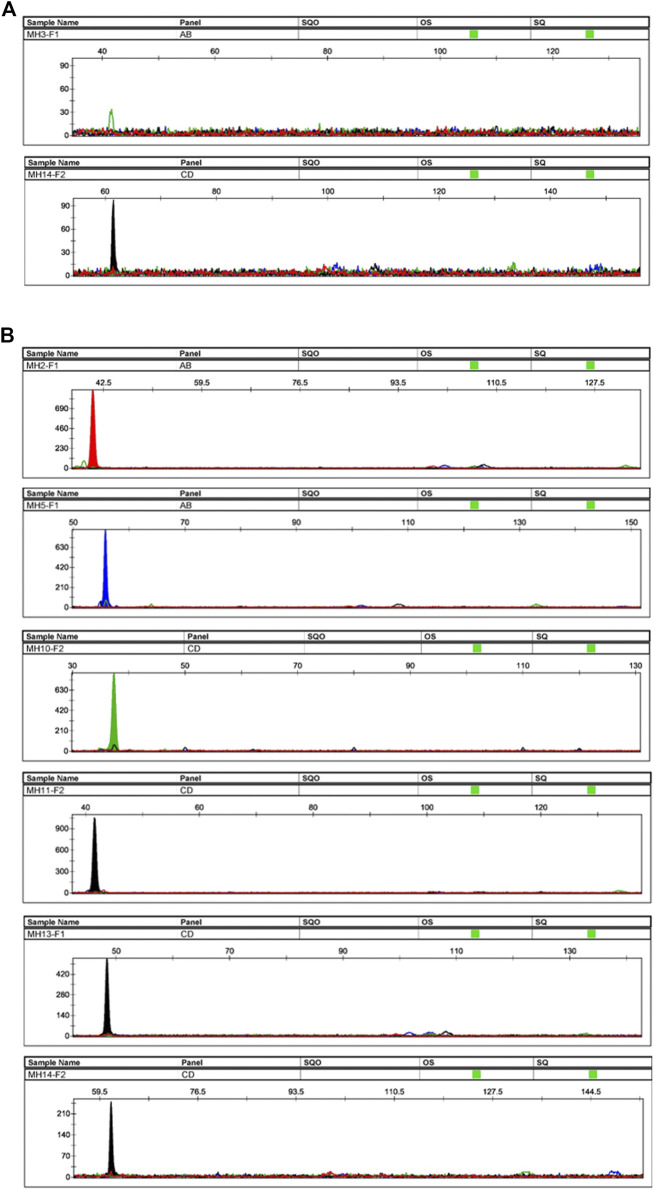
Plasma samples’ profiles. **(A)** Profiles of the plasma sample from family No. 2. The sample had two informative alleles, but only MH14-F2 displayed a positive result. The peak height of MH3-F1 did not reach the threshold, and the fetal DNA was not successfully genotyped. **(B)** Profiles of plasma sample of family No. 6. The sample had six informative alleles, and all loci displayed positive results.

For each family, after genotyping of the reference parents and amniotic fluid, at least one informative marker was obtained (except for the absence of informative markers for No. 24). The allele-specific primers of these informative markers were used to amplify cffDNA in the maternal plasma. Four families displayed one informative allele (No. 15, 16, 20, 25), seven families displayed two informative alleles (No. 2, 3, 10, 14, 17, 19, 23), four families displayed three informative alleles (No. 8, 9, 12, 18), six families displayed four informative alleles (No. 1, 4, 11, 13, 22, 26), one family displayed five informative alleles (No. 21), and three families displayed six informative alleles (No. 5, 6, 7). All informative alleles obtained positive results, except for MH3-F1 in family No. 2. This might due to the low concentration of cfDNA samples (non-detectable at <0.1 ng/μL) (Figure 6A). Taking the plasma samples from the No. 6 family as an example, six informative alleles were successfully genotyped (Figure 6B). The length of all detected fetal fragments ranged from 60 to 150 bp (ARMS-PCR amplicons), which was consistent with previously reported cffDNA size distribution (Zhang et al., 2020). All fetal alleles detected in maternal plasma were double confirmed by genotyping the gDNA of the father and the amniotic fluid.

### 3.6 Correlation Between Cell-Free DNA Concentration and Detection Rate

Since the concentration of fetal DNA is difficult to obtain, we used the concentration of cfDNA extracted from the maternal plasma as a reference. According to previous reports, cffDNA accounts for 5–20% of the total cfDNA (Scotchman et al., 2019). Table 1 shows the extracted cfDNA concentration, the expected and observed number of informative markers of cffDNA. The cfDNA concentration was measured using the Qubit™ dsDNA HS kit ranged from 0.156 to 1.43 ng/μL (the sample from family No. 2 was below the 0.01 ng/μL detection threshold). Therefore, based on the theoretical ratio of 5–20%, the concentration of cffDNA was distributed approximately between 0.0078 and 0.286 ng/μL. In previous sensitivity determinations, the detection limit of most markers was 0.025 ng (Zhang et al., 2020). Therefore, to improve the detection rate, the number of cycles in the ARMS-PCR and SBE steps was increased to 32. Figure 6A showed that cffDNA could be amplified in maternal plasma even below 0.01 ng/μL (MH14-F2 in family No. 2). In addition, the results suggested that the cfDNA concentration and cffDNA detection rate had no obvious correlation.

**TABLE 1:** The concentration of the cell-free DNA (cfDNA).

Family ID	Concentration (ng/μL)	Expected number of informative markers^a^	Observed number of informative markers^b^
1	0.388	4	4
2	Out of range	2	1
3	0.754	2	2
4	0.204	4	4
5	0.266	6	6
6	0.414	6	6
7	0.716	6	6
8	0.63	3	3
9	0.2	3	3
10	0.696	2	2
11	0.632	4	4
12	0.538	3	3
13	1.03	4	4
14	1.03	2	2
15	1.12	1	1
16	0.82	1	1
17	1.115	2	2
18	1.43	3	3
19	1.14	2	2
20	0.764	1	1
21	0.606	5	5
22	0.224	4	4
23	0.54	2	2
24	0.392	0	0
25	0.156	1	1
26	0.884	4	4
Total		77	76

a The expected number of informative markers was obtained from the reference parent and the amniotic fluid genotype.

b The number of observed informative markers referred to the successful detection of fetal DNA, in maternal plasma.

### 3.7 Size Distribution of Informative Markers’ Amplicons

Table 2 showed the information of all primers used to detect cffDNA in this study. The ARMS-PCR amplicons of these primers observed in previous reports were 60–150 bp, and the SBE amplicons were 32–79 bp (Zhang et al., 2020). The size of all amplicons makes the detection of cffDNA highly feasible because the median length of cffDNA is only 143 bp. The frequency of lengths exceeding 300 bp was <1%. In this study, SBE amplicons of cffDNA detected by the information marker were 26–71 bp, with an average of 45 bp. In general, the effectiveness of primers can be roughly divided into two categories. Primer MH3-F1 failed to target cffDNA in one of the maternal plasmas. For the remaining primers, all samples showed positive results.

**TABLE 2:** Primer information of informative markers and detected fetal DNA size.

Primers	Successful/Total samples	Detection rate	ARMS-PCR amplicons (bp)	SBE amplicons (bp)	Observed size of cffDNA (bp)
MH1-F1	1/1	1	134	26	34
MH2-F1	4/4	1	108	32	41
MH2-F2	1/1	1	108	32	41
MH3-F1	1/2	0.5	121	36	42
MH3-F2	3/3	1	121	36	41
MH4-F1	5/5	1	101	43	46
MH4-F2	1/1	1	101	43	46
MH5-F1	4/4	1	60	52	56
MH5-F2	1/1	1	60	52	56
MH6-F1	5/5	1	114	61	64
MH6-F2	1/1	1	114	61	64
MH7-F1	1/1	1	90	65	67
MH7-F2	4/4	1	90	65	67
MH8-F1	7/7	1	126	71	77
MH8-F2	3/3	1	126	71	77
MH9-F1	1/1	1	150	27	35
MH9-F2	1/1	1	150	27	33
MH10-F1	2/2	1	106	31	39
MH10-F2	5/5	1	106	31	38
MH11-F1	1/1	1	69	35	43
MH11-F2	6/6	1	69	35	42
MH12-F2	2/2	1	141	39	44
MH13-F1	6/6	1	95	43	48
MH14-F2	8/8	1	79	58	61
MH15-F1	1/1	1	113	63	68
MH15-F2	1/1	1	113	63	68
Average			106	45	51

### 3.8 Performance Comparison of SNP-SNPs, DIP-STRs, and SNP-STRs

In this study, the 15 SNP-SNPs previously reported can be used to detect cffDNA in maternal plasma. Previous studies have shown that when the minor DNA is 0.025 ng, SNP-SNPs, DIP-STRs, and SNP-STRs all yield positive results (Zhang et al., 2020). Most SNP-SNPs targeted minor DNA in 1:1,000 mixtures, DIP-STRs targeted a ratio of 1:1,000 (Tan et al., 2017b), and SNP-STRs targeted a ratio of 1:100 (Tan et al., 2018). A total of 6 DIP-STRs and 11 SNP-STRs detected cffDNA in the maternal plasma. The specificity of SNP-STR primers was lower than that of DIP-STR. The detection rates were 42.9 and 64.1%, respectively (Tan et al., 2021). Although the specificity of SNP-SNP primers is less than DIP-STR, cffDNA accounts for 5–20% of the total cfDNA in maternal blood. Thus, cffDNA can theoretically still be detected. Figure 5 and Table 1 showed the expected and observed numbers of informative markers of SNP-SNPs. There were 77 informative markers in all 26 families. The cffDNA was successfully detected in 76 of them, with a detection rate of 98.7%.

## 4 Discussion

This study demonstrates that SNP-SNPs markers can be applied to detect UDM and cffDNA through CE-based ARMS-PCR and SNaPshot technology. Compared with MPS (Bennett et al., 2019; Chen et al., 2019), the traditional CE platform saves time, effort, and expense, and the analysis is less complicated. Therefore, it can be performed in most forensic laboratories.

SNP-SNPs used ARMS-PCR to target minor DNA in unbalanced mixtures. The premise is that the minor DNA has a unique SNP1 allele that differs from the major components. Thus, when the major and minor DNA are oppositely homozygous for SNP1, or the major DNA is SNP1 homozygous and the minor DNA is SNP1 heterozygous, the minor DNA can be distinguished. However, those with the same SNP1 genotypes are not feasible (Figure 1). Our previous research demonstrated that the panel of 15 SNP-SNPs had several strengths, such as good polymorphism, high identification ability, high potential for mixture detection, no gender restriction, and wide distribution in the genome. These advantages strongly support the value of the SNP-SNP panel for various forensic applications (Zhang et al., 2020).

In general, the actual environment of forensic cases is harsh. Particularly due to the high temperature, sample degradation commonly occurred (Machida et al., 2017). We simulated the degraded samples in the laboratory during incubation at different time. The detection results of the Agilent High Sensitivity DNA Kit and commercial STR kit reflected the degree of DNA degradation (Supplementary Figure S2). The traditional methods of analyzing degraded DNA include short amplicon markers, such as mini-STRs, insertion or deletion of bases in the genomes (i.e., INDELs), multiple SNP-microhaplotypes (Kidd et al., 2017; Chen et al., 2018), and increasing sensitivity of PCR reactions. However, these strategies can only obtain the informative genotype of degraded single-source DNA. As a result, minor DNA in UDM is not detected. The amplificons of the 15 SNP-SNPs in this study only involved 60–150 bp. The ARMS-PCR method guarantees primer amplification specificity to a certain extent and is theoretically suitable for UDM analysis.

This study validates the application potential of SNP-SNPs in UDM: (1) The detection sensitivity of minor DNA in degraded mixtures was high, and the input DNA reached 0.05 to 0.025 ng; (2) Minor DNA was sensitively detected in the degraded mixtures at a 1:1,000 dilution; (3) Degraded single or mixed DNA can be analyzed to obtain a complete genotype. The present findings indicate that the conventional DNA amount (0.1–10 ng) required for forensic case analysis can be achieved. Based on the CE platform, SNP-SNPs are a unique and powerful DNA tool. In the future, we can consider using the MPS platform to increase the detection throughput after the MPS platform is widely used and the cost of sequencing comes down.

NIPT is a better method compared to invasive prenatal testing. The emotional and physical stress of pregnant women can be lessened, and the difficulty of sampling can be reduced. Although MPS has a high throughput, it is time-consuming, laborious, costly, and requires experienced data analysts. In addition, current research towards cffDNA detection is still in the exploratory stage. Preliminary shows that MPS combines microhaplotypes and mature SNPs and STR kit has certain application potential in NIPPT (Bai et al., 2020; Ou and Qu 2020; Ou and Bai 2021; Shen et al., 2021). In this study, based on the CE platform, the 15 SNP-SNPs, provide a new and cost-effective strategy for NIPPT. Allele-specific primers can easily distinguish alleles that are not shared with the mother from the maternal plasma. Our study is a preliminary exploration of whether this method can be used to amplify maternal plasma cffDNA. The results confirm the feasibility of this strategy.

The SNP-SNP microhaplotype features short amplification, extensive polymorphism, and wide distribution in the genome. Thus, it is an ideal marker for NIPPT. The cffDNA is usually 100–200 bp in length (Lo et al., 2010). The amplicons of 15 SNP-SNPs are 60–150 bp, which fully meets the prerequisites for length. All 26 cffDNA samples used at least one informative marker to achieve a positive result, except for MH3-F1 in the No. 2 family, which was not genotyped correctly. This may be due to the low concentration of the cfDNA sample, which was below detection limit. The detected fetal DNA fragments ranged from 26 to 71 bp. Even when the free DNA concentration was <0.01 ng/μL, positive results were obtained (MH14-F2). We assumed that the main reason for the negative results was the low primer specificity or the low quantity of target DNA fragments. In addition, the cause of non-specific peaks suggested that the specificity of primers was not high enough. For MH2-F1, a noise peak near the target peak was observed, but it did not interfere the accurate genotype of cffDNA. The number of informative loci in 16 families was less than three, indicating the need to develop markers with higher specific primers and the inclusion of more samples from different pregnancy periods for more comprehensive and systematic research and analysis.

Among the 26 cffDNA samples in this study, the positive detection rate of SNP-SNPs was approximately 98.7% (76/77). In the 21 previously studied samples (Tan et al., 2021), the positive detection rate of DIP-STRs was approximately 64.1% (24/39), and that of SNP-STRs was approximately 42.9% (15/35). The detection rate of SNP-SNPs was higher than that of DIP-STRs and SNP-STRs, which may be due to the higher specificity of SNP-SNP primers and shorter amplicons. However, more samples need to be studied to verify this hypothesis. A prior study used 28 DIP-STR markers to detect 48 cffDNA samples (Amandine and Diana 2018). However, the authors only selected one informative marker for each detection sample. Thus, comprehensive information on the detection efficiency of all markers was lacking. In this study, we used all the potentially informative markers displayed by parental genotypes to detect cffDNA and comprehensively analyzed the results. In addition, we found that not all potential informative markers can target the cffDNA of a specific maternal plasma. However, our method limits the detection of minor DNA only when SNP 1 is homozygous in the maternal SNP-SNP microhaplotype, and then the corresponding SNP 2 will provide some information. This means that only a part of informative alleles of the 15 SNP-SNPs will be detected, which may not be enough to determine paternity in practice. This reminds us that more polymorphic microhaplotype markers with higher Ae values will be needed in the future (Bai et al., 2020), or that it is necessary to develop multiple markers for a target sequence and apply them jointly to improve identification capabilities.

## 5 Conclusion

In summary, based on the CE platform, ARMS-PCR combined with SNaPshot technology to detect SNP-SNPs can help overcome the challenge of unbalanced degraded two-person DNA mixtures in forensic cases, regardless of gender composition. SNP-SNPs can be used as a powerful supplement to DIP-STRs, SNP-STRs, and DIP-SNPs. In the future, a combination of multiple genetic markers may provide more valuable information for the interpretation of mixtures. Moreover, we also proved that this method can be applied to target cffDNA in maternal plasma, providing a new and cost-effective strategy for NIPPT. We can further optimize and expand this research. First, more sensitive and specific primers can be designed to improve the success rate of cffDNA detection. Second, additional polymorphic microhaplotype markers with higher Ae values can improve the identification performance of the paternity test system. The MPS platform can be harnessed to construct an appropriate mathematical model to calculate the paternity power of the analysis system. In addition, samples from different periods during pregnancy need to be collected and tested for a more comprehensive analysis.

## Data Availability

The datasets presented in this study can be found in online repositories. The names of the repository/repositories and accession number(s) can be found in the article/Supplementary Material. https://doi.org/10.6084/m9.figshare.16863262.v2.

## Abbreviations

Ae, effective number of alleles; ARMS, amplification-refractory mutation system; CE, capillary electrophoresis; MPS, massively parallel sequencing; NIPPT, noninvasive prenatal paternity testing; NIPT, noninvasive prenatal testing; SBE, single base extension; UDM, unbalanced and degraded mixture.
