# Corrigendum: Set of 15 SNP-SNP markers for detection of unbalanced degraded DNA mixtures and noninvasive prenatal paternity testing

**DOI:** 10.3389/fgene.2025.1553186

**Published:** 2025-01-21

**Authors:** Ranran Zhang, Yu Tan, Li Wang, Hui Jian, Jing Zhu, Yuanyuan Xiao, Mengyu Tan, Jiaming Xue, Fan Yang, Weibo Liang

**Affiliations:** ^1^ Department of Forensic Genetics, West China School of Basic Medical Sciences and Forensic Medicine, Sichuan University, Chengdu, China; ^2^ Department of Obstetrics and Gynecology, West China Second University Hospital, Key Laboratory of Birth Defects and Related Diseases of Women and Children of Ministry of Education, Sichuan University, Chengdu, China; ^3^ Department of Forensic Science and Technology, Sichuan Police College, Luzhou, China; ^4^ Department of Ultrasonography, West China Second University Hospital Sichuan University, Chengdu, China

**Keywords:** SNP-SNP microhaplotype, amplification-refractory mutation system PCR, SNaPshot, unbalanced and degraded mixture, cell-free fetal DNA, noninvasive prenatal paternity testing

In the published article, an **author name** was incorrectly written as “Rangran Zhang”. The correct spelling is “Ranran Zhang.”

The authors apologize for this error and state that this does not change the scientific conclusions of the article in any way. The original article has been updated.

