# Supplementary material for: Set of 15 SNP-SNP Markers for Detection of Unbalanced Degraded DNA Mixtures and Noninvasive Prenatal Paternity Testing
Source: Front Genet. 2022 Feb 10;12:800598. doi: 10.3389/fgene.2021.800598 (PMC8866868; doi:10.3389/fgene.2021.800598)
Supplement: Supplementary file 1 [file DataSheet1.docx]

Supplementary Material


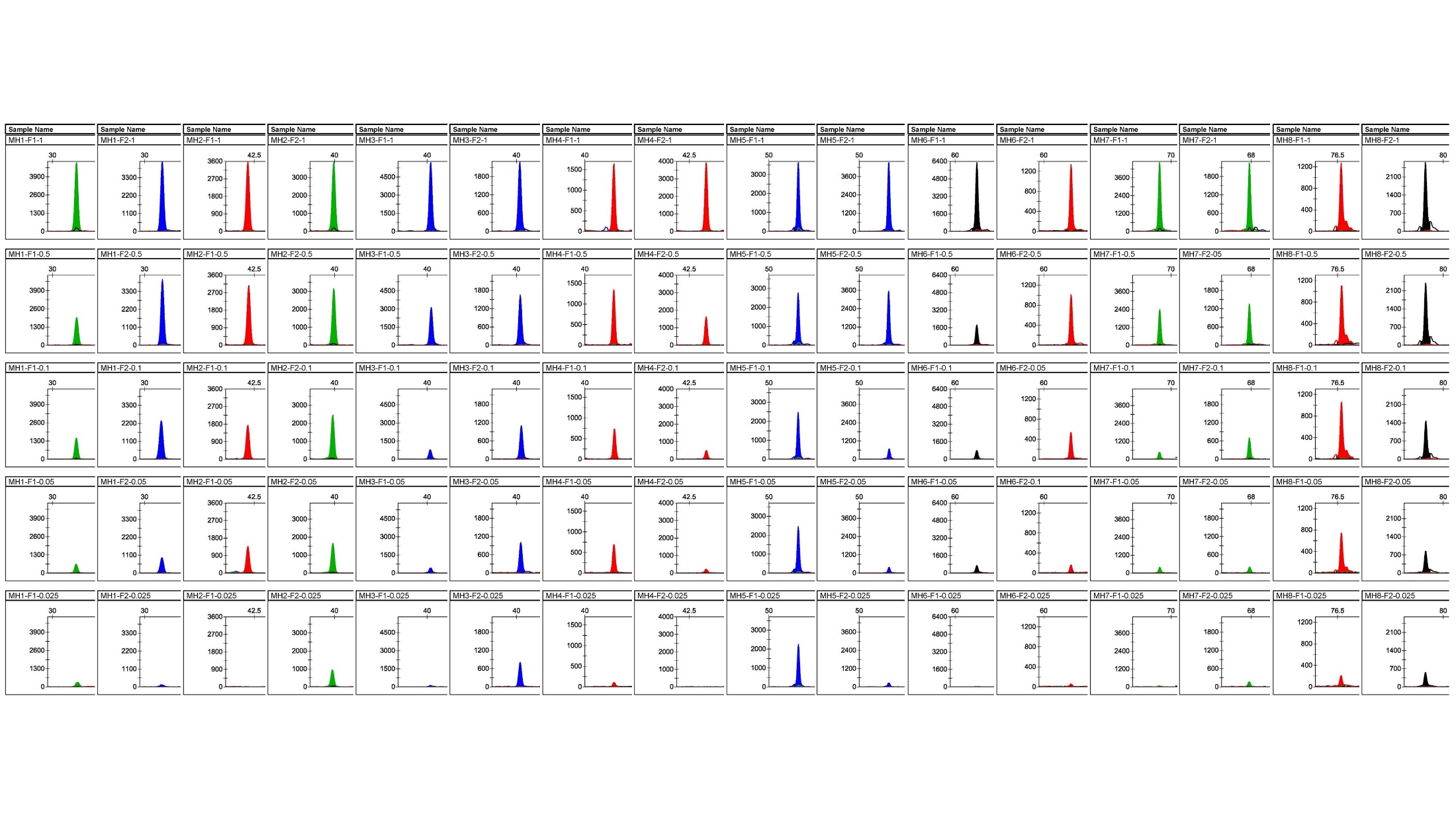


**(A)**


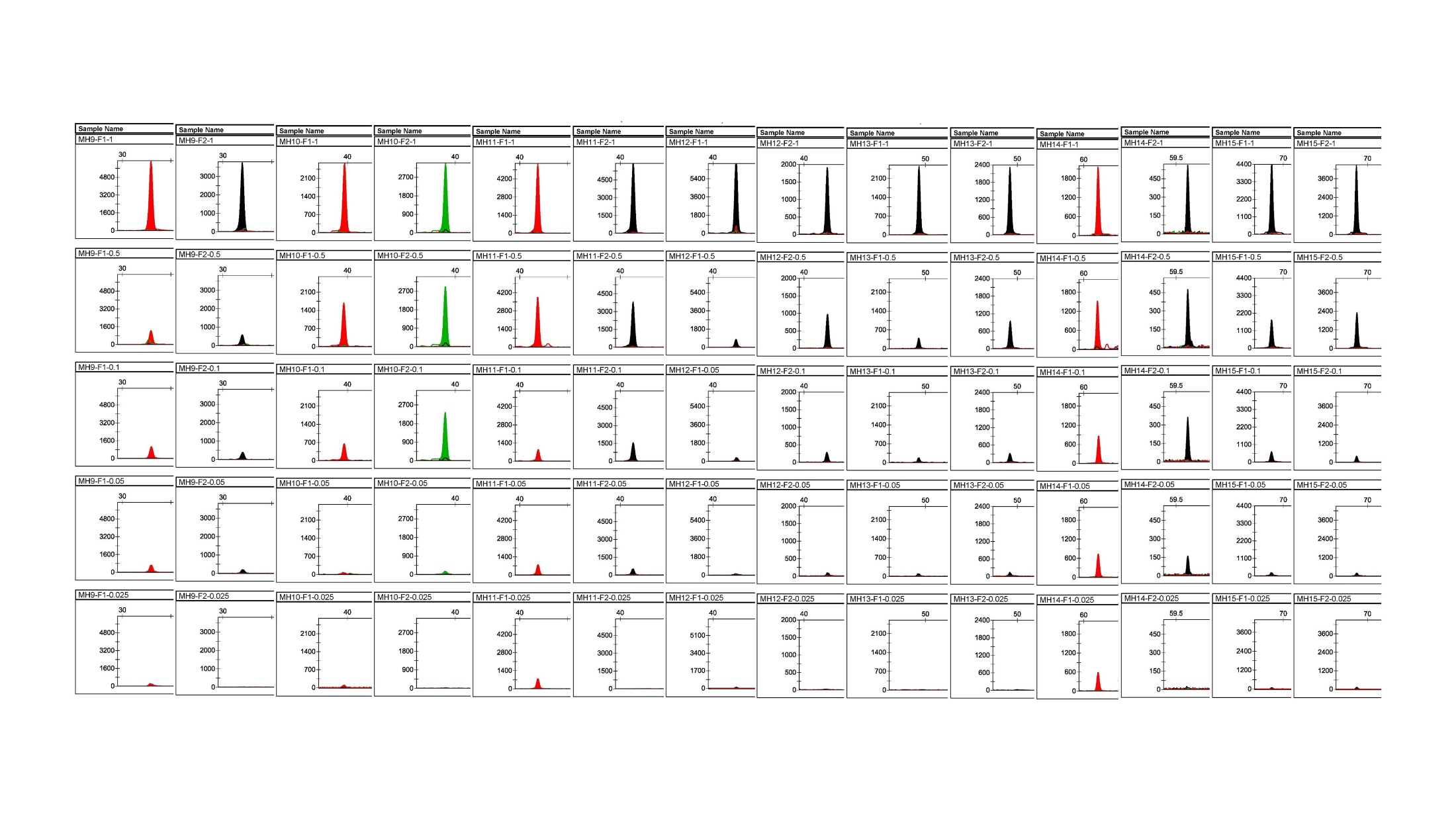


**(B)**

**Supplementary Figure 1.** Sensitivity profiles of a single allele-specific primer. (A) and (B) represent the two panels constructed previously, containing 8 (MH1~MH8) and 7 (MH9~MH15) loci respectively (see our previous study (Zhang et al., 2020) for more details). Each locus had two allele-specific primers, F1 and F2. All 30 primers showed a positive result of 0.025 ng, except MH2-F1, MH4-F2, MH7-F1, MH9-F2, MH10-F2, MH11-F2, MH12-F2, MH13-F1/F2, and MH14-F2, which had a detection sensitivity of 0.05 ng.


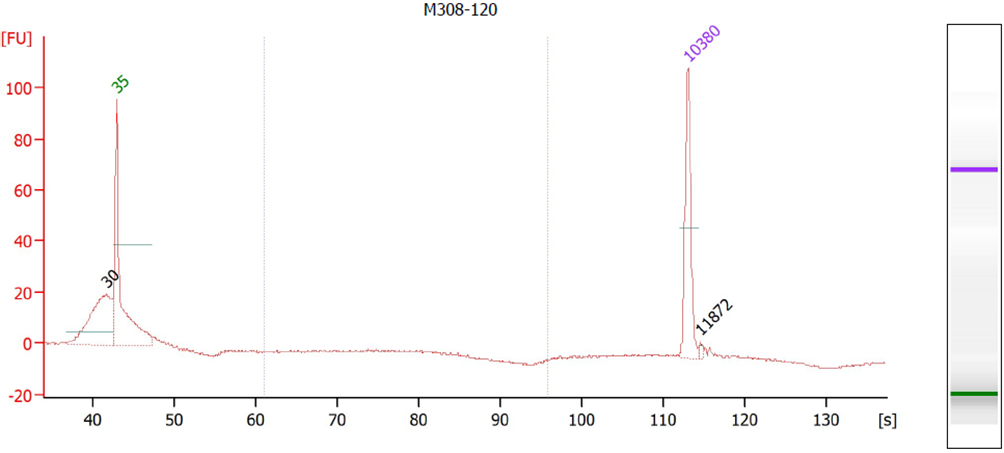


**（A）**


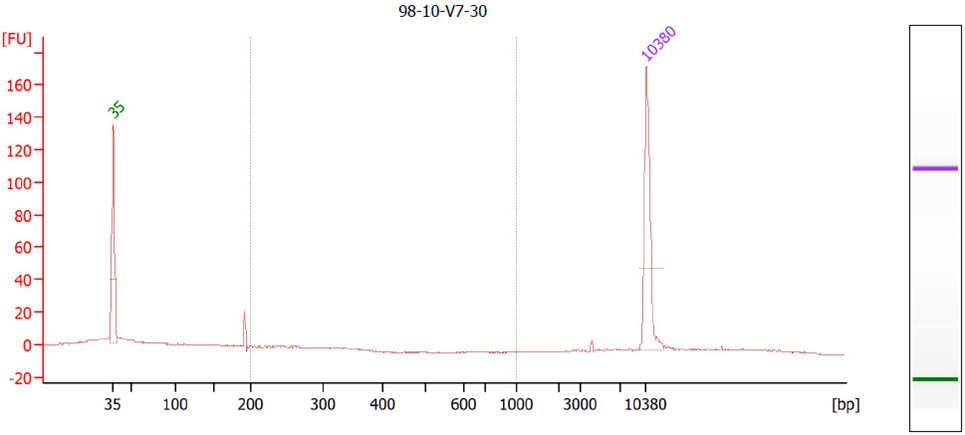


**（B）**


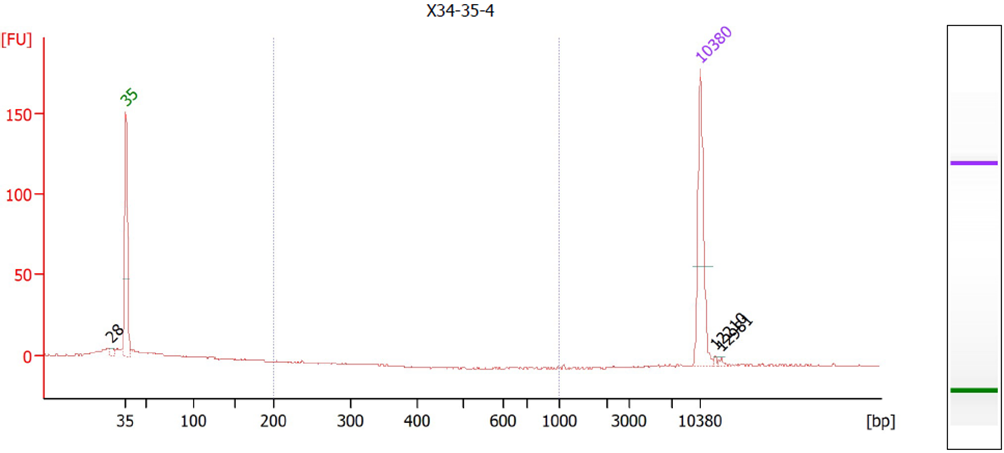


**（C）**

**Supplementary Figure 2.** Evaluation of the degree of fragmentation of degraded samples. A High Sensitivity DNA Kit was used on the Agilent 2100 Bioanalyzer. **(A)** The fragment range of the standard DNA M308 concentrated at approximately 30bp after incubation at 98°C for 120 min. **(B)** The fragment range of sample V7 concentrated at approximately 200bp after incubation at 98°C for 30 min. **(C)** The fragment range of sample X34 concentrated at approximately 28bp after incubation at 98°C for 35 min. The input concentration of the DNA was 5 ng/uL.


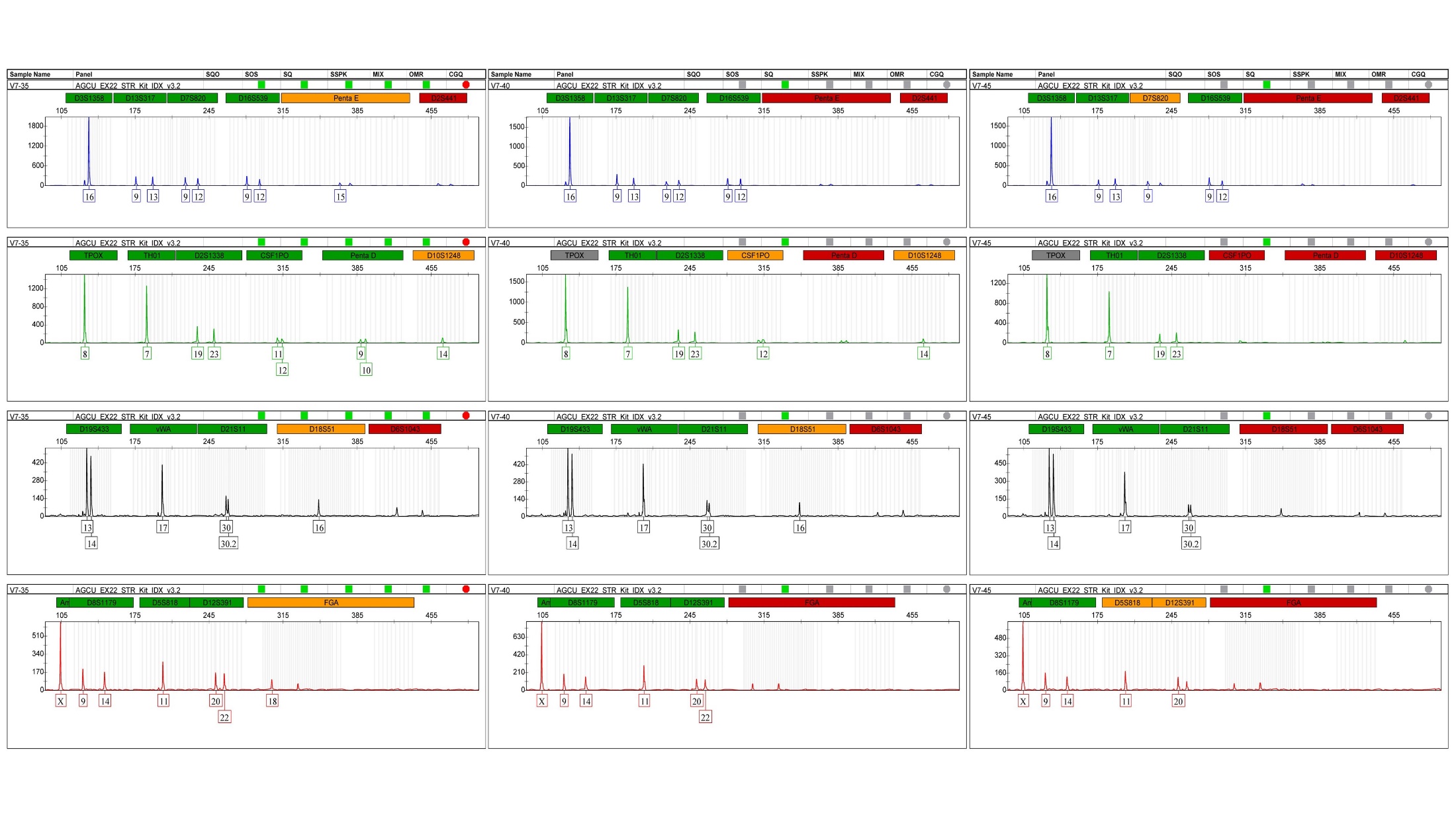


**（A）**


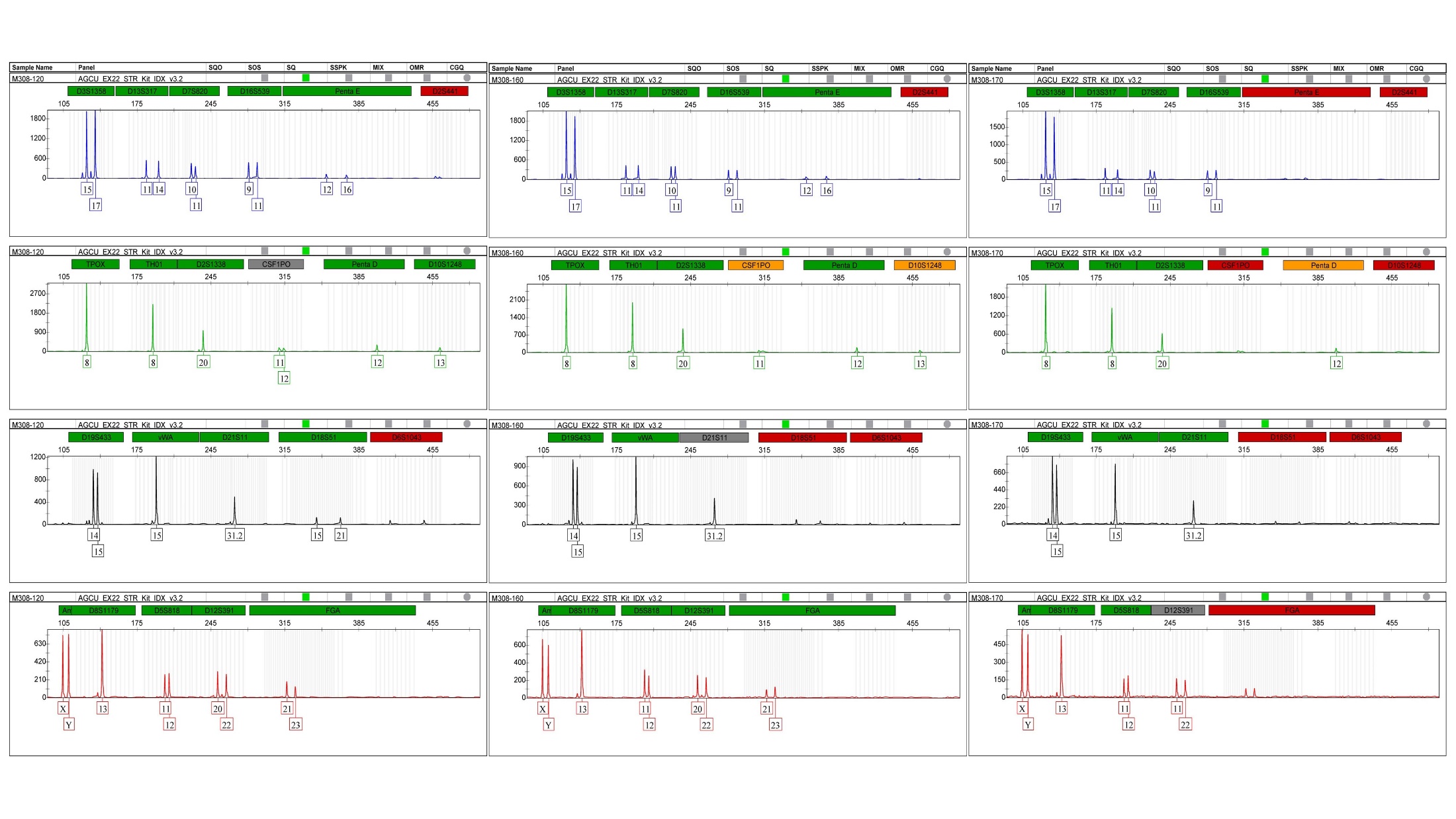


**（B）**


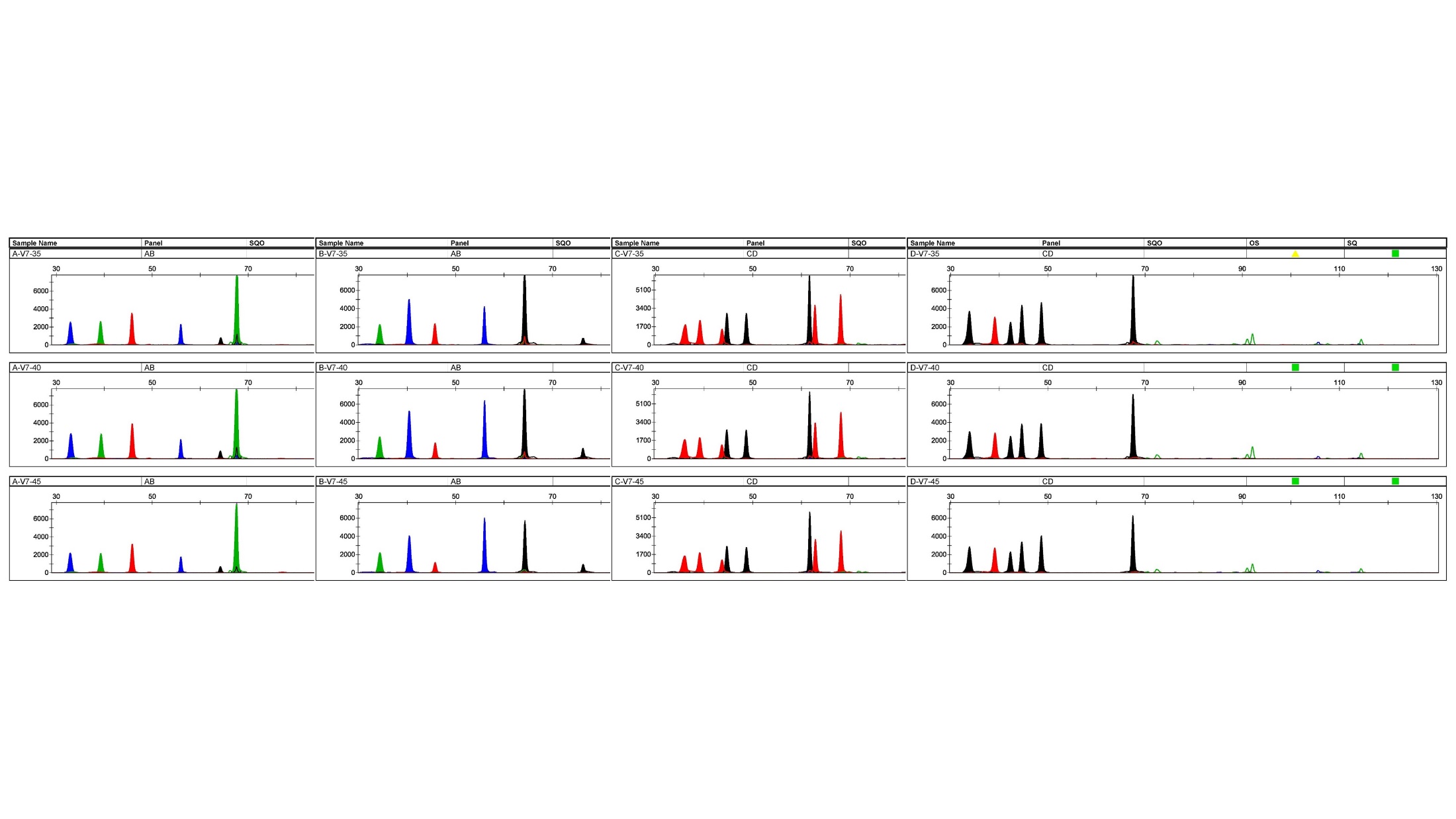


**（C）**


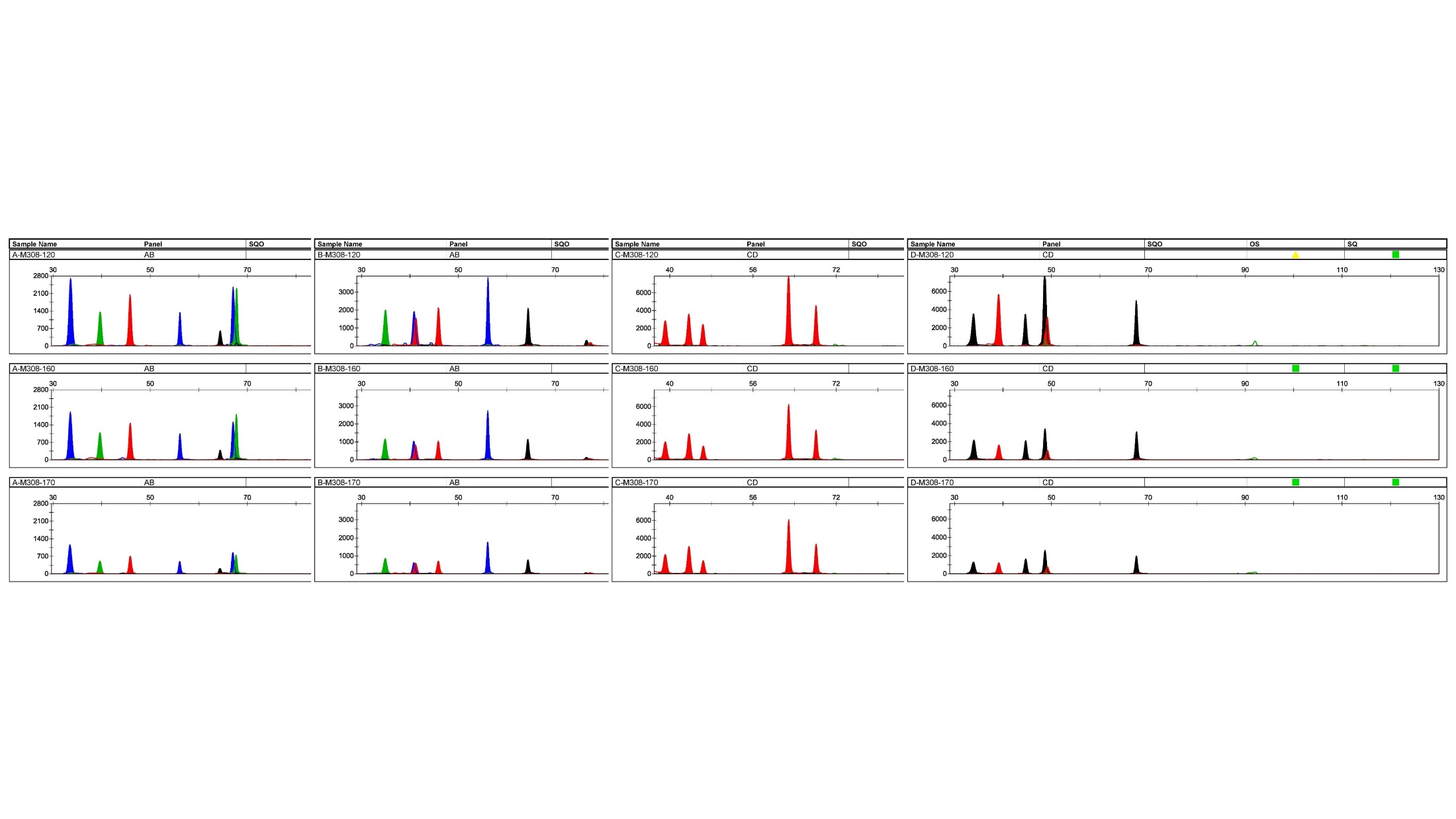


**（D）**

**Supplementary Figure 3.** Profiles of degraded single-source DNA. DNA V7 obtained from a randomly selected individual was processed at 98°C for 35, 40, 45 min. Standard DNA M308 was processed at 98°C for 120, 160, 170 min. **(A and B)** Profiles obtained using the commercial STR kit at 35, 40, and 45 min from left to right. **(A)** For V7, drop-out of the STR Kit began at approximately 385bp at 35 min and 280bp at 40 and 45min. **(B)** For M308, drop-out of the STR Kit began at approximately 385bp at 120 min and 280bp at 160 and 170 min. Only a subset of the loci of the two samples were genotyped at different degradation times. **(C and D)** Profiles obtained using the SNP-SNP multiplex system at 35, 40, and 45 min from top to bottom. All loci of the two samples were successfully genotyped at the different degradation times.


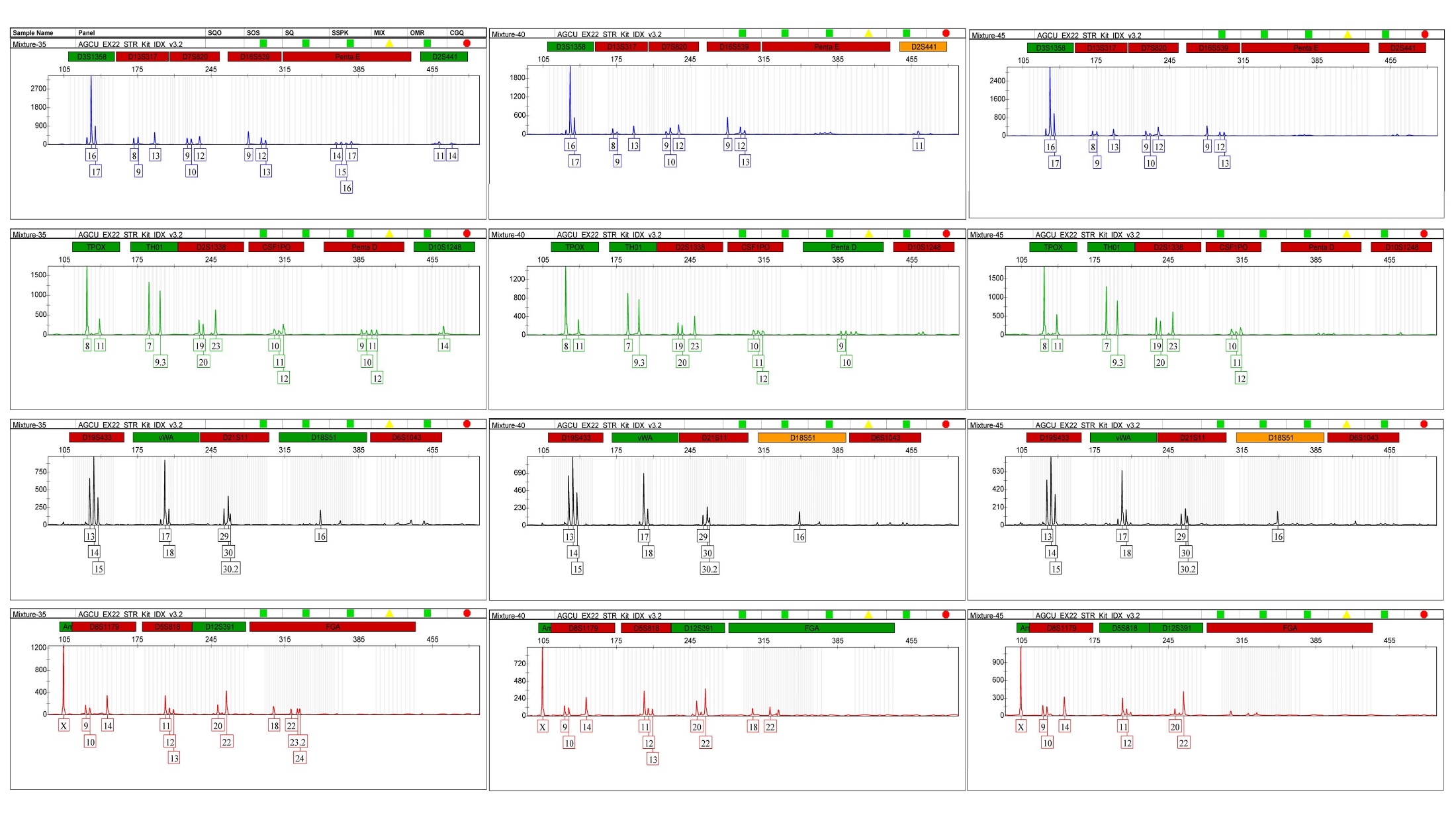


**（A）**


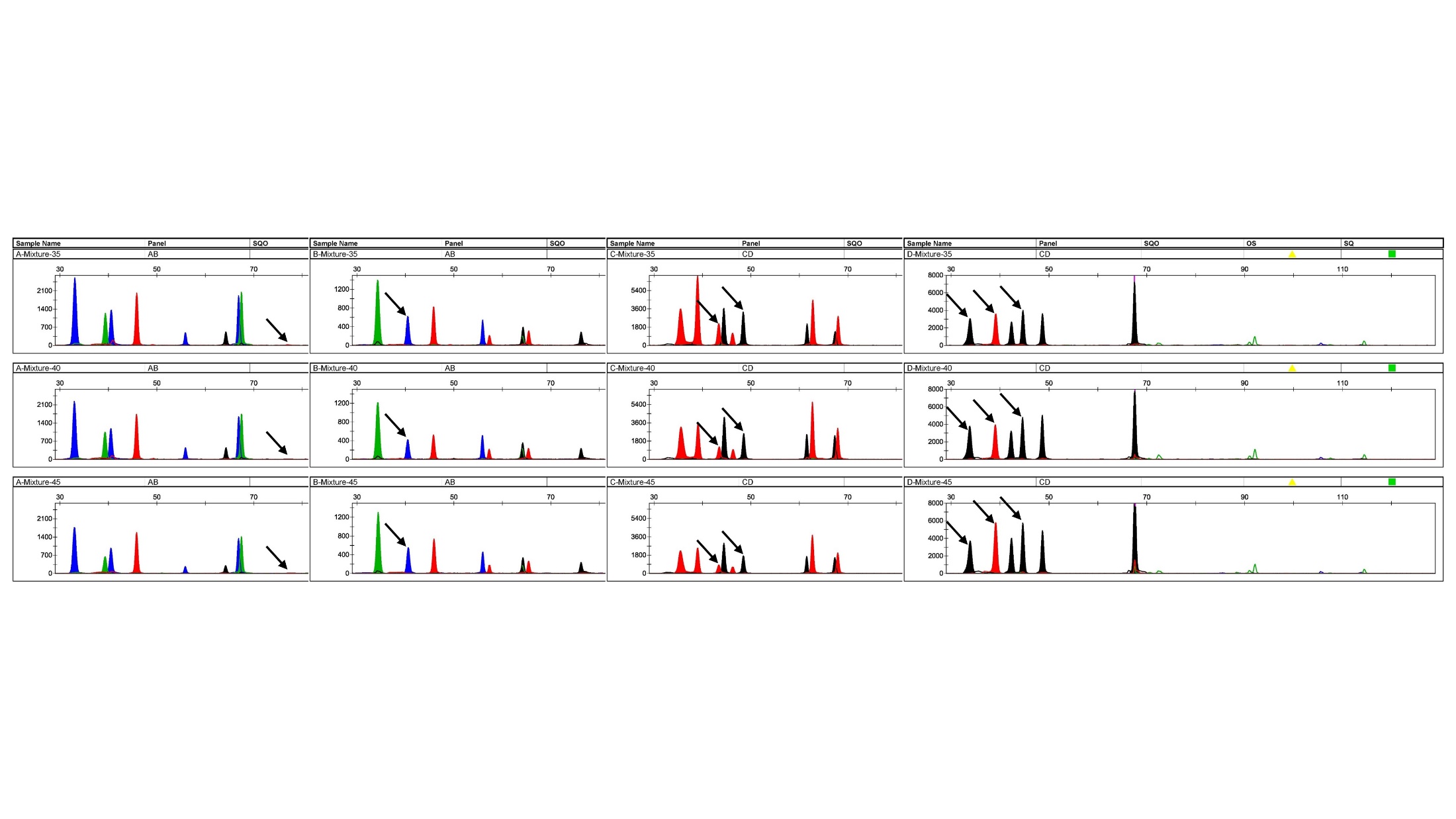


**（B）**

**Supplementary Figure 4.** Profiles of the degraded mixture. The mixture was processed at 98°C for 35, 40, and 45 min. **(A)** Profiles obtained at 30, 40, and 45 min from left to right using the commercial STR kit. The drop-out of the STR Kit began at approximately 310bp at 35 min, 280bp at 40 min, and 180bp at 45 min. **(B)** Profiles obtained at 30, 40, and 45 min from top to bottom using the SNP-SNP multiplex system. All loci in the sample were successfully genotyped at the different degradation times. The black arrows indicated informative markers in the mixture.

| **Supplement Table 1**. Description of SNP-SNPs included in this study^1^. | | | | |  |
| --- | --- | --- | --- | --- | --- |
| ID | SNP-SNP | SNP1 | Chr | Location（GRCh37/hg19) | Extent in bp |
| MH1 | rs10445426-rs57907290 | G/T | 18 | 12195773-12195803 | 30 |
| MH2 | rs12101725-rs55649144 | A/G | 15 | 92575365-92575386 | 21 |
| MH3 | rs3843625-rs12422436 | C/T | 12 | 58858690-58858715 | 25 |
| MH4 | rs9938522-rs9940690 | A/T | 16 | 13552242-13552251 | 9 |
| MH5 | rs3109851-rs6848611 | C/T | 4 | 26473621-26473631 | 10 |
| MH6 | rs468851-rs468852 | C/T | 22 | 29956137-29956156 | 19 |
| MH7 | rs2527748-rs2527749 | G/T | 8 | 5396199-5396225 | 26 |
| MH8 | rs35443929-rs6462431 | A/C | 7 | 32935434-32935439 | 5 |
| MH9 | rs12950438-rs12950190 | C/T | 17 | 59846353-59846363 | 10 |
| MH10 | rs10119697-rs10961215 | C/G | 9 | 13741227-13741252 | 24 |
| MH11 | rs59588112-rs8018285 | C/T | 14 | 25768631-25768641 | 10 |
| MH12 | rs6663660-rs9426355 | C/T | 1 | 29774262-29774287 | 25 |
| MH13 | rs2012094-rs220181 | C/T | 21 | 43561440-43561468 | 28 |
| MH14 | rs7724803-rs6554864 | C/T | 5 | 14950999-14951005 | 6 |
| MH15 | rs68012481-rs2498233 | C/T | 6 | 23007491-23007496 | 5 |
| 1 More details of the 15 SNP-SNPs are available in our previous study (Zhang et al., 2020) | | | | |  |

| **Supplementary Table 2**. Characteristics of the donors. | | | | | |
| --- | --- | --- | --- | --- | --- |
| Family ID | Sample ID | Origin | Gestation Week | Concentration^1^ | Clinical state |
| No.1 | K181203-9102 | Mother’s peripheral blood | ＞18 | 15.96 | No special |
|  | K181203-9103 | Father’s peripheral blood |  | 5.58 |  |
|  | K181119-7023 | Amniotic fluid |  | 29.6 |  |
|  | K181203-9102 | Maternal plasma |  | 0.388 |  |
| No.2 | K181203-9104 | Mother’s peripheral blood | ＞18 | 3.6 | No special |
|  | K181203-9105 | Father’s peripheral blood |  | 5.3 |  |
|  | K181119-7046 | Amniotic fluid |  | 12.4 |  |
|  | K181203-9104 | Maternal plasma |  | Out of range |  |
| No.3 | K181225-9126 | Mother’s peripheral blood | ＞18 | 16.96 | No special |
|  | K181225-9127 | Father’s peripheral blood |  | 5.86 |  |
|  | K181207-7512 | Amniotic fluid |  | 8 |  |
|  | K181225-9126 | Maternal plasma |  | 0.754 |  |
| No.4 | K190312-9162 | Mother’s peripheral blood | ＞18 | 51.24 | No special |
|  | K190312-9163 | Father’s peripheral blood |  | 16.21 |  |
|  | K190225-0520 | Amniotic fluid |  | 2.92 |  |
|  | K190312-9162 | Maternal plasma |  | 0.204 |  |
| No.5 | K190312-9164 | Mother’s peripheral blood | ＞18 | 43.87 | No special |
|  | K190312-9165 | Father’s peripheral blood |  | 5.13 |  |
|  | K190226-0534 | Amniotic fluid |  | 37 |  |
|  | K190312-9164 | Maternal plasma |  | 0.266 |  |
| No.6 | K190312-9166 | Mother’s peripheral blood | ＞18 | 63.24 | No special |
|  | K190312-9167 | Father’s peripheral blood |  | 40.59 |  |
|  | K190226-0537 | Amniotic fluid |  | 9.86 |  |
|  | K190312-9166 | Maternal plasma |  | 0.414 |  |
| No.7 | K190312-9168 | Mother’s peripheral blood | ＞18 | 8.83 | No special |
|  | K190312-9169 | Father’s peripheral blood |  | 10.54 |  |
|  | K190226-0387 | Amniotic fluid |  | 11.8 |  |
|  | K190312-9168 | Maternal plasma |  | 0.716 |  |
| No.8 | K190312-9170 | Mother’s peripheral blood | ＞18 | 37.78 | No special |
|  | K190312-9171 | Father’s peripheral blood |  | 13.14 |  |
|  | K190226-0609 | Amniotic fluid |  | 5.2 |  |
|  | K190312-9170 | Maternal plasma |  | 0.63 |  |
| No.9 | K190312-9180 | Mother’s peripheral blood | ＞18 | 12.64 | No special |
|  | K190312-9181 | Father’s peripheral blood |  | 21.98 |  |
|  | K190226-0762 | Amniotic fluid |  | 5.94 |  |
|  | K190312-9180 | Maternal plasma |  | 0.2 |  |
| No.10 | K190312-9189 | Mother’s peripheral blood | ＞18 | 28.04 | No special |
|  | K190312-9190 | Father’s peripheral blood |  | 35.45 |  |
|  | K190226-0872 | Amniotic fluid |  | 5.58 |  |
|  | K190312-9189 | Maternal plasma |  | 0.696 |  |
| No.11 | K190312-9191 | Mother’s peripheral blood | ＞18 | 23.66 | No special |
|  | K190312-9192 | Father’s peripheral blood |  | 13.06 |  |
|  | K190226-0930 | Amniotic fluid |  | 7.58 |  |
|  | K190312-9191 | Maternal plasma |  | 0.632 |  |
| No.12 | K190312-9193 | Mother’s peripheral blood | ＞18 | 18.72 | No special |
|  | K190312-9194 | Father’s peripheral blood |  | 32.66 |  |
|  | K190226-0956 | Amniotic fluid |  | 31.2 |  |
|  | K190312-9193 | Maternal plasma |  | 0.538 |  |
| No.13 | K190102-9130 | Mother’s peripheral blood | ＞18 | 40.48 | No special |
|  | K190102-9131 | Father’s peripheral blood |  | 12.38 |  |
|  | K181217-7600 | Amniotic fluid |  | 14.8 |  |
|  | K190102-9130 | Maternal plasma |  | 1.03 |  |
| No.14 | K190102-9138 | Mother’s peripheral blood | ＞18 | 29.86 | No special |
|  | K190102-9139 | Father’s peripheral blood |  | 23.92 |  |
|  | K190117-0161 | Amniotic fluid |  | 3.3 |  |
|  | K190102-9138 | Maternal plasma |  | 1.03 |  |
| No.15 | K190305-9152 | Mother’s peripheral blood | ＞18 | 33.51 | No special |
|  | K190305-9153 | Father’s peripheral blood |  | 31.18 |  |
|  | K190211-0307 | Amniotic fluid |  | 27.19 |  |
|  | K190305-9152 | Maternal plasma |  | 1.12 |  |
| No.16 | K190305-9154 | Mother’s peripheral blood | ＞18 | 39.75 | No special |
|  | K190305-9155 | Father’s peripheral blood |  | 28.11 |  |
|  | K190214-0364 | Amniotic fluid |  | 19.39 |  |
|  | K190305-9154 | Maternal plasma |  | 0.82 |  |
| No.17 | K190305-9156 | Mother’s peripheral blood | ＞18 | 17.06 | No special |
|  | K190305-9157 | Father’s peripheral blood |  | 10.93 |  |
|  | K190212-0326 | Amniotic fluid |  | 7.12 |  |
|  | K190305-9156 | Maternal plasma |  | 1.115 |  |
| No.18 | K190306-9158 | Mother’s peripheral blood | ＞18 | 45.84 | No special |
|  | K190306-9159 | Father’s peripheral blood |  | 53.99 |  |
|  | K190219-0426 | Amniotic fluid |  | 7.98 |  |
|  | K190306-9158 | Maternal plasma |  | 1.43 |  |
| No.19 | K190306-9160 | Mother’s peripheral blood | ＞18 | 43 | No special |
|  | K190306-9161 | Father’s peripheral blood |  | 22.11 |  |
|  | K190219-0431 | Amniotic fluid |  | 14.3 |  |
|  | K190306-9160 | Maternal plasma |  | 1.14 |  |
| No.20 | K190416-9210 | Mother’s peripheral blood | ＞18 | 22.61 | No special |
|  | K190416-9211 | Father’s peripheral blood |  | 36.39 |  |
|  | K190328-1128 | Amniotic fluid |  | 8.57 |  |
|  | K190416-9210 | Maternal plasma |  | 0.764 |  |
| No.21 | K90416-9212 | Mother’s peripheral blood | ＞18 | 23.23 | No special |
|  | K90416-9213 | Father’s peripheral blood |  | 21.53 |  |
|  | K190329-1159 | Amniotic fluid |  | 15.9 |  |
|  | K90416-9212 | Maternal plasma |  | 0.606 |  |
| No.22 | K190416-9214 | Mother’s peripheral blood | ＞18 | 17.48 | No special |
|  | K190416-9217 | Father’s peripheral blood |  | 43.64 |  |
|  | K190401-1169 | Amniotic fluid |  | 7.42 |  |
|  | K190416-9214 | Maternal plasma |  | 0.224 |  |
| No.23 | K190416-9215 | Mother’s peripheral blood | ＞18 | 23.85 | No special |
|  | K190416-9216 | Father’s peripheral blood |  | 74.66 |  |
|  | K190315-0880 | Amniotic fluid |  | 34.8 |  |
|  | K190417-9215 | Maternal plasma |  | 0.54 |  |
| No.24 | K1904239220 | Mother’s peripheral blood | ＞18 | 19.55 | No special |
|  | K1904239221 | Father’s peripheral blood |  | 25.56 |  |
|  | K190408-1320 | Amniotic fluid |  | 7.76 |  |
|  | K1904239220 | Maternal plasma |  | 0.392 |  |
| No.25 | K1904239224 | Mother’s peripheral blood | ＞18 | 50.14 | No special |
|  | K1904239225 | Father’s peripheral blood |  | 20.51 |  |
|  | K190410-1412 | Amniotic fluid |  | 14.91 |  |
|  | K1904239224 | Maternal plasma |  | 0.156 |  |
| No.26 | K181203-ZGR-B | Mother’s peripheral blood | ＞18 | 45.8 | No special |
|  | K181203-LL | Father’s peripheral blood |  | 79.4 |  |
|  | K181126-7222 | Amniotic fluid |  | 36 |  |
|  | K181203-ZGR | Maternal plasma |  | 0.884 |  |

1 Peripheral blood from the mother and father and amniotic fluid from the mother were quantified using a NanoDrop™ 1000 spectrophotometer. Maternal plasma was quantified using a Qubit 3.0 fluorometer.

| **Supplementary Table 3**. Informative markers' details of each family. | | | | | | | | | | | | | | | | |
| --- | --- | --- | --- | --- | --- | --- | --- | --- | --- | --- | --- | --- | --- | --- | --- | --- |
| Family ID | rs10445426-rs57907290 | | rs12101725-rs55649144 | | rs3843625-rs12422436 | | rs9938522-rs9940690 | | rs3109851-rs6848611 | | rs468851-rs468852 | | rs2527748-rs2527749 | | rs35443929-rs6462431 | |
|  | (G-)^1^ | (T-)^2^ | (A-) | (G-) | (C-) | (T-) | (A-) | (T-) | (C-) | (T-) | (C-) | (T-) | (G-) | (T-) | (A-) | (C-) |
| No.1 |  |  |  |  |  |  |  |  |  |  |  |  |  |  | A |  |
| No.2 |  |  |  |  | T |  |  |  |  |  |  |  |  |  |  |  |
| No.3 |  |  |  |  |  |  |  |  |  |  |  |  |  |  | A |  |
| No.4 | A^3^ |  | T |  |  | C |  |  |  |  |  |  |  |  |  |  |
| No.5 |  |  |  |  |  | C |  |  | C |  |  |  |  | T | A |  |
| No.6 |  |  | T |  |  |  |  |  | C |  |  |  |  |  |  |  |
| No.7 |  |  |  |  |  |  | T |  |  | C | C |  |  |  | A |  |
| No.8 |  |  |  |  |  |  |  |  |  |  | C |  |  |  |  |  |
| No.9 |  |  | T |  |  |  |  |  |  |  |  |  |  |  | A |  |
| No.10 |  |  |  |  |  |  |  | T |  |  |  |  |  |  | A |  |
| No.11 |  |  |  | T |  |  |  |  |  |  | C |  |  |  |  |  |
| No.12 |  |  |  |  |  |  |  |  | C |  | C |  |  |  | A |  |
| No.13 |  |  |  |  |  |  | T |  |  |  |  |  |  |  |  |  |
| No.14 |  |  |  |  |  |  |  |  |  |  |  |  |  |  |  |  |
| No.15 |  |  |  |  |  |  |  |  |  |  |  |  |  |  |  |  |
| No.16 |  |  |  |  |  |  |  |  |  |  |  |  |  |  |  |  |
| No.17 |  |  |  |  | T |  |  |  |  |  |  |  |  |  |  |  |
| No.18 |  |  |  |  |  |  |  |  |  |  |  |  |  | T |  | A |
| No.19 |  |  | T |  |  |  |  |  |  |  |  |  | T |  |  |  |
| No.20 |  |  |  |  |  |  |  |  |  |  |  |  |  |  |  |  |
| No.21 |  |  |  |  |  |  | T |  |  |  |  |  |  | T |  | A |
| No.22 |  |  |  |  |  |  | T |  |  |  | C |  |  |  |  |  |
| No.23 |  |  |  |  |  |  |  |  |  |  |  | CT |  |  |  | G |
| No.24 |  |  |  |  |  |  |  |  |  |  |  |  |  |  |  |  |
| No.25 |  |  |  |  |  |  | T |  |  |  |  |  |  |  |  |  |
| No.26 |  |  |  |  |  | C |  |  | C |  |  |  |  | T |  |  |
| Number of informative markers per primer | 1 | 0 | 4 | 1 | 2 | 3 | 5 | 1 | 4 | 1 | 5 | 1 | 1 | 4 | 7 | 3 |

**Continued:**

| **Supplementary Table 3**. Informative markers' details of each family. | | | | | | | | | | | | | | |
| --- | --- | --- | --- | --- | --- | --- | --- | --- | --- | --- | --- | --- | --- | --- |
| Family ID | rs12950438-rs12950190 | | rs10119697-rs10961215 | | rs59588112-rs8018285 | | rs6663660-rs9426355 | | rs2012094-rs220181 | | rs7724803-rs12521912 | | rs68012481-rs2498233 | |
|  | (C-)^1^ | (T-)^2^ | (C-) | (G-) | (C-) | (T-) | (C-) | (T-) | (C-) | (T-) | (C-) | (T-) | (C-) | (T-) |
| No.1 | A^3^ |  |  |  |  |  |  |  |  |  |  | G |  | G |
| No.2 |  |  |  |  |  |  |  |  |  |  |  | G |  |  |
| No.3 |  |  | T |  |  |  |  |  |  |  |  |  |  |  |
| No.4 |  |  |  |  |  |  |  |  |  |  |  | G |  |  |
| No.5 |  |  |  |  |  |  |  | G | G |  |  |  |  |  |
| No.6 |  |  |  | A |  | G |  |  | G |  |  | G |  |  |
| No.7 |  | G |  |  |  |  |  |  | G |  |  |  |  |  |
| No.8 |  |  |  | A |  | G |  |  |  |  |  |  |  |  |
| No.9 |  |  |  |  |  |  |  |  |  |  |  |  | A |  |
| No.10 |  |  |  |  |  |  |  |  |  |  |  |  |  |  |
| No.11 |  |  |  |  |  | G |  |  |  |  |  | G |  |  |
| No.12 |  |  |  |  |  |  |  |  |  |  |  |  |  |  |
| No.13 |  |  |  | A |  |  |  | G | G |  |  |  |  |  |
| No.14 |  |  |  |  |  | G |  |  |  |  |  | G |  |  |
| No.15 |  |  |  |  | A |  |  |  |  |  |  |  |  |  |
| No.16 |  |  |  |  |  |  |  |  | G |  |  |  |  |  |
| No.17 |  |  | T |  |  |  |  |  |  |  |  |  |  |  |
| No.18 |  |  |  |  |  |  |  |  | G |  |  |  |  |  |
| No.19 |  |  |  |  |  |  |  |  |  |  |  |  |  |  |
| No.20 |  |  |  | T |  |  |  |  |  |  |  |  |  |  |
| No.21 |  |  |  |  |  | G |  |  |  |  |  | G |  |  |
| No.22 |  |  |  |  |  | G |  |  |  |  |  | G |  |  |
| No.23 |  |  |  |  |  |  |  |  |  |  |  |  |  |  |
| No.24 |  |  |  |  |  |  |  |  |  |  |  |  |  |  |
| No.25 |  |  |  |  |  |  |  |  |  |  |  |  |  |  |
| No.26 |  |  |  | T |  |  |  |  |  |  |  |  |  |  |
| Number of informative markers per primer | 1 | 1 | 2 | 5 | 1 | 6 | 0 | 2 | 6 | 0 | 0 | 8 | 1 | 1 |

1 and 2 represent the two alleles of SNP1 at each locus; 3 represents the SNP2 genotype at each locus. Note that only when SNP1 has an ARMS-PCR amplification product does SNP2 have a corresponding SBE extension product. At this time, the corresponding SNP1-SNP2 is a single-stranded haplotype.
